# Advanced nanofiber therapy: multifunctional silver-nanoparticles@polyacrylonitrile incorporating *Syzygium guineense* extracts for enhanced *in vivo* diabetic wound-healing and robust antimicrobial defense

**DOI:** 10.1039/d5na00343a

**Published:** 2025-08-22

**Authors:** Teshale Ayano Begeno, Yaqi Zhang, Abdurohman Mengesha Yessuf, Tibebu Shiferaw Kassa, Ahmed M. Salama, Weiguo Wang, Zhenxia Du

**Affiliations:** a College of Chemistry, Beijing University of Chemical Technology Beijing 100029 China ayanoteshale@gmail.com duzx@mail.buct.edu.cn; b Beijing Key Laboratory of Advanced Functional Polymer Composites, College of Materials Science and Engineering, Beijing University of Chemical Technology Beijing 100029 China; c Department of Orthopaedics, China-Japan Friendship Hospital Beijing 100029 China

## Abstract

Green-synthesized silver nanoparticles (Bio-Ag NPs) derived from *Syzygium guineense* offer an eco-friendly, cost-effective platform with potent antibacterial activity and biocompatibility. These nanoparticles were integrated into electrospun polyacrylonitrile (PAN) nanofibers, creating Bio-Ag NPs@PAN nanocomposites for enhanced diabetic wound healing applications. The synthesized materials were systematically characterized using Fourier-transform infrared spectroscopy (FTIR), X-ray diffraction (XRD), scanning electron microscopy (SEM), and transmission electron microscopy (TEM). The antibacterial efficacy of Bio-Ag NPs was evaluated against Gram-positive *Staphylococcus aureus* and Gram-negative *Escherichia coli*, demonstrating inhibition zones of 17.0 ± 0.310 mm and 16.3 ± 0.290 mm, respectively. Additionally, the antioxidant potential of Bio-Ag NPs was confirmed using the DPPH assay, highlighting their physiological benefits. *In vivo* studies on a diabetic rat revealed the remarkable wound-healing efficiency of Bio-Ag NPs@PAN nanofibers. Over 3, 7, 11, and 14 days, these nanofibers significantly enhanced wound closure by promoting re-epithelialization, fibroblast proliferation, and extracellular matrix formation. Notably, Bio-Ag NPs(B)@PAN nanofibers accelerated diabetic wound healing by 52%, 68%, 88%, and 99% on days 3, 7, 11, and 14, respectively, with increased collagen deposition. This study demonstrates the multifunctional capabilities of Bio-Ag NPs@PAN nanofibers in addressing the challenges associated with diabetic wound healing, offering faster recovery and improved wound closure. Furthermore, the findings underscore the potent antioxidant and antibacterial properties of Bio-Ag NPs, emphasizing their potential for diverse biomedical applications.

## Abstract 2

Green-synthesized *S. guineense* Bio-Ag NPs exhibit synergistic antibacterial activity against *S. aureus* and *E. coli* pathogens and antioxidant properties. Bio-Ag NPs@PAN nanofibers accelerate diabetic wound healing *in vivo*.

## Graphical abstract

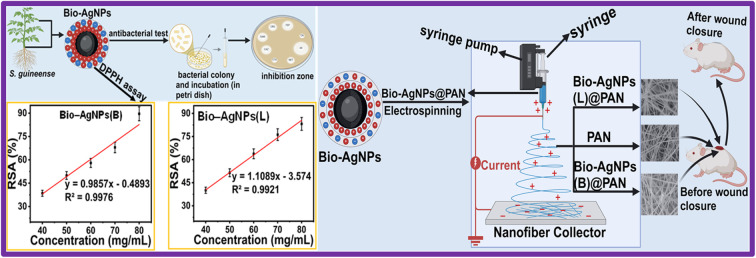

## 1 Introduction

Bio-synthesized nanoparticles (Bio-NPs) are being widely investigated in various biomedical fields as a means of enhancing and safeguarding our health.^1^ The world is now focusing more on Bio-NPs because of their high compatibility, low toxicity, cost-effectiveness, environmental friendliness, and efficient production *via* green synthesis for producing genuine and reasonably priced metal nanoparticles for various biological applications.^2^ Green-synthesized nanoparticles offer an eco-friendly, cost-effective, and less toxic alternative to conventional chemical synthesis, being derived from plant extracts of leaves, flowers, fruits, and stem bark.^3,4^ There are diverse types of metal nanoparticles such as gold nanoparticles (AuNPs),^5–8^ silver nanoparticles (Ag NPs),^9–12^ Platinum nanoparticles (Pt NPs),^13–16^ palladium nanoparticles (Pd NPs),^17–19^ copper nanoparticles(Cu NPs)^20–23^ and others that can exhibit different characteristics depending on their size, shape, and composition. Among these, biosynthesized silver nanoparticles (Bio-Ag NPs) are promising candidates for antibacterial and antioxidant applications due to their dual functionalities, stability, biocompatibility, and versatility in biomedical applications.^11,24^

Plants are natural biochemical factories, historically and currently used in medicine, producing potent bioactive compounds (phytochemicals) rich in active ingredients within all their parts.^25^ The widely grown fruit-bearing tree *Syzygium guineense* (*S. guineense*) (Willd.) DC. produces a substantial amount of agricultural byproducts, including leaves, stem bark, leftover fruits, charcoal, and lumber. It is a traditional medicinal herb that is also referred to as wild berry, waterberry, and snake bean.^26^*S. guineense* is a medium-sized or tall evergreen tree that ranges in height from 15 to 30 meters. It belongs to the Myrtaceae family of plants and is found in portions of Africa and Asia.^27,28^*S. guineense* has demonstrated potent antibacterial properties against a wide range of pathogens, including both Gram-positive (G +ve) and Gram-negative (G −ve) bacteria. This efficacy is attributed to its bioactive compounds, such as flavonoids, tannins, and essential oils, which possess strong antimicrobial activity.^26,27^ This medicinal plant is used to treat urinary tract infections, skin infections, hypertension, malaria, snake bites, hemorrhoids, gonorrhea, tuberculosis (TB), stomach aches, liver problems, cardiovascular disorders, and diabetes, which can all be treated using an infusion made from its leaves, fruits, and bark.^29–32^ While a few studies have explored the antibacterial properties of bio-synthesized nanoparticles derived from the leaves of this medicinal plant,^33^ there is a notable lack of research on its potential for diabetic wound healing and antioxidant activity. This study aims to address this gap by comprehensively investigating the plant's efficacy in promoting diabetic wound healing and its antioxidant capabilities, thereby expanding the understanding of its therapeutic applications and potential benefits in managing diabetic complications.

Diabetes mellitus (DM) is one of the most prevalent chronic diseases globally, affecting more than 500 million individuals. It is characterized by a disruption in glucose metabolism, leading to elevated blood sugar levels.^34,35^ Beyond its primary metabolic implications, diabetes often results in severe complications, such as chronic diabetic wounds and diabetic foot ulcers. These conditions not only impair quality of life but also significantly increase the risk of limb amputation and mortality, highlighting the urgent need for effective therapeutic interventions to manage and mitigate these life-threatening complications.^36,37^ The healing process in diabetic wounds is greatly impaired due to a complex inflammatory environment characterized by bacterial infections, excessive accumulation of reactive oxygen species (ROS), and hypoxic conditions. As a result, diabetic wounds heal much slower than normal wounds.^38^ The delayed healing of diabetic wounds is primarily driven by hyperglycemia, which leads to a cascade of complications, including impaired keratinocyte and fibroblast activity, disrupted epithelialization, and hindered wound closure. These factors collectively contribute to the prolonged and often ineffective healing process in diabetic wounds.^39–41^ In recent years, plant-derived bio-silver nanoparticles (Bio-Ag NPs) have garnered significant attention in the field of wound healing, particularly for diabetic wounds. This is due to their multifaceted therapeutic properties, including antibacterial, anti-inflammatory, and regenerative effects. Additionally, Bio-Ag NPs exhibit reduced toxicity, enhanced biocompatibility, potent antioxidant activity, and the ability to stimulate fibroblast proliferation and collagen synthesis.^42,43^

Polyacrylonitrile (PAN) is a synthetic polymer with potential in wound healing due to its biocompatibility, versatility, and ability to be processed into fibers and nanofibers.^44^ PAN-based materials promote wound healing through cell proliferation, moisture retention, antibacterial properties, biodegradability, and mechanical support.^44–46^ When combined with nanoparticles, PAN not only reduces the risk of wound infection but also mimics the mechanical strength of the extracellular matrix, providing a supportive structure for tissue regeneration. Its properties can be further enhanced by incorporating natural materials such as silver nanoparticles (Ag NPs), which improve its functionality for faster and more effective wound healing. This synergistic combination leverages the antimicrobial and regenerative capabilities of Ag NPs while maintaining the structural integrity and biocompatibility of PAN, making it a promising material for advanced wound care applications.^47–50^

Phyto-mediated synthesis is a highly efficient, green, one-step method for creating Ag NPs. Plant phytochemicals naturally reduce silver ions (Ag^+^ to Ag^0^*via* redox reactions), stabilize the resulting nanoparticles, and conserve the energy of biosynthesis while eliminating synthetic toxicants.^51,52^ Ag NPs exhibit strong antibacterial properties, excellent biocompatibility, and the ability to enhance tissue regeneration and cell proliferation, making them a highly promising tool for wound healing.^53–55^ Additionally, Ag NPs possess antioxidant properties, reduce inflammation, and prevent infections, further underscoring their therapeutic potential.^56–58^ To optimize their effectiveness in diabetic wound healing, Ag NPs can be incorporated into electrospun nanofibers, creating a synergistic combination that enhances wound-healing processes.^59,60^ Owing to these remarkable properties, this study investigated the antibacterial and antioxidant capabilities of bio-synthesized silver nanoparticles (Bio-Ag NPs) derived from *S. guineense*, specifically targeting *E. coli* and *S. aureus*. Additionally, the Bio-Ag NPs were incorporated into PAN nanofibers and evaluated *in vivo* for their potential to enhance diabetic wound healing. The study focused on leveraging the reactive oxygen species (ROS) scavenging ability of Bio-Ag NPs to address the impaired healing processes in diabetic wounds, offering a novel and effective approach to managing this challenging ailment.

## 2 Materials and methods

### 2.1. Chemicals

#### 2.1.1. Solvents

Methanol (MeOH, ≥99.9%) and acetonitrile (ACN, ≥99.9%) were both supplied by ANPEL Laboratory Technologies (Shanghai) Inc.; ethanol (EtOH, ≥99.7%) was supplied by Tianjin ZhiYuan Reagent Co., Ltd; deionized water (di-H_2_O, high purity) was provided by Tianjin Guanla Power Supply Co., Ltd; dimethyl sulfoxide (DMSO, ≥99.9%) was acquired from Shanghai Nianxing Industrial Co., Ltd, and *N*,*N*-dimethylformamide (DMF, ≥99%) was procured from Tianjin Science Bio-Tech Co., Ltd.

#### 2.1.2. Reagents

Silver nitrate (AgNO_3_, ≥99.8%) was obtained from Shanghai Zoran New Material Co., Ltd; polyacrylonitrile (PAN) with a molecular weight of 150 000 was procured from Tianjin Science Bio-Tech Co., Ltd, and Shanghai Aladdin Biochemical Technology, China, supplied the 2,2-diphenyl-1-picrylhydrazyl (DPPH) used in this study. All chemicals were of analytical grade and used directly without further purification.

### 2.2. Plant material collection and pre-treatment

The leaves and stem bark of an *S. guineense* plant were collected from shero kebele Misha Wereda, Hadiya Zone, Central Ethiopia region. The place is around 320 kilometers from Ethiopia's capital city, Addis Ababa. The plant materials were identified by expert taxonomist Melaku Wondafrash and deposited as voucher specimens at the National Herbarium (ETH), Addis Ababa University, Ethiopia.^61–63^ The plant samples were washed and chopped into small pieces and allowed to dry in a shaded area to prevent direct exposure to sunlight. They were then dispersed and frequently turned over to avoid fermentation and degradation. This was done for nearly four weeks. Using an electric grinder, the dried materials were crushed into a fine powder, then placed in polyethylene bags with labeling on them, and kept at room temperature for further analysis.

### 2.3. Extraction

**Scheme 1:**
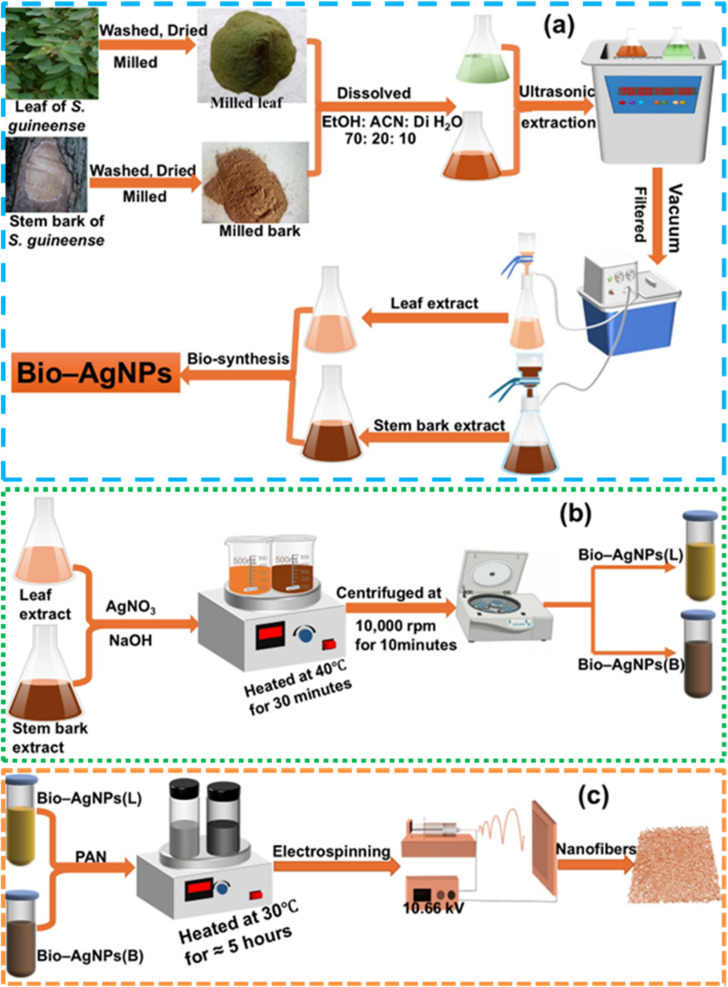
(a) Ultrasonic-assisted extraction of *S. guineense* leaf (L) and stem bark (B), (b) bio-synthesis of silver nanoparticles from leaf extract (Bio-Ag NPs(L)) and stem bark extract (Bio-Ag NPs(B)), and (c) schematic representation of the electrospinning process for fabricating Bio-Ag NPs@PAN.

Extraction in pharmaceuticals involves using solvents to separate active plant-based compounds from inactive ones, using techniques to eliminate inert materials and isolating therapeutically active components.^25,64^ Ultrasonic-assisted extraction (UAE) exploits acoustic cavitation to rapidly enhance solute dissolution, diffusional mass transfer, and thermal equilibration, thereby optimizing extraction yields.^64,65^ In this study, 25 g of milled leaf and stem bark of *S. guineense* were extracted by the UAE technique at 70 °C for 1 hour using a solvent system containing ethanol (140 mL), acetonitrile (40 mL), and deionized water (20 mL) in a total volume of 200 mL (70 : 20 : 10). The resultant extracts were then subjected to vacuum filtering, as illustrated in Scheme 1a.

### 2.4. Synthesis of Bio-Ag NPs

A 3.5 g sample of AgNO_3_ was dissolved in 400 mL of deionized water and dispersed into two distinct 500 mL beakers. Each beaker was filled with 100 mL of *S. guineense* leaf (L) and stem bark (B) extracts, which acted as encapsulating, stabilizing, reducing, and capping agents. To adjust the pH (11), a 0.5 M NaOH solution was added dropwise under constant stirring. NaOH's alkaline conditions enhance the phytochemical reducing power, accelerate metal ion reduction (Ag^+^ → Ag^0^), and promote uniform nanoparticle formation, leading to smaller, less aggregated nanoparticles with increased surface area. Thus, the majority of the particles generated at low pH values (7, 8, and 8.5) have irregular shapes. However, the Ag NPs produced at high pH values (10 and 11) are smaller and more uniform.^66,67^ The reaction mixture was heated to 40 °C and stirred using a magnetic stirrer for 1 hour. Subsequently, the mixture was incubated in a dark environment at room temperature for 24 hours to expedite bio-silver nanoparticle synthesis, and a color change indicates the creation of the Bio-Ag NPs. The synthesized Bio-Ag NPs were then rinsed repeatedly with deionized water to eliminate any impurities. The purified nanoparticles were subsequently centrifuged at 10 000 rpm for 10 minutes to increase purity. Finally, the Bio-Ag NPs were dried at 60 °C for around 2 hours, minced into fine powder, and kept for further characterization and analysis. The biosynthesis process of Bio-Ag NPs is illustrated in Scheme 1b.

### 2.5. Fabrication of Bio-Ag NPs@Polyacrylonitrile (PAN) and PAN nanofibers

PAN and Bio-Ag NPs@PAN nanofibers were synthesized through the electrospinning technique. In CYKY's CY-SPLF-ES1 model single syringe pump, the speed and flow rate can be adjusted to achieve custom fabrication of fibers. Three distinct solutions were prepared: 10 wt% (w/w) PAN, 10 wt% (w/w) Bio-Ag NPs(L)@PAN, and 10 wt% (w/w) Bio-Ag NPs(B)@PAN. These solutions were thoroughly dissolved in dimethylformamide (DMF) by stirring in vials at 30 °C for approximately five hours at 400 rpm. The resulting homogeneous solutions were then loaded into a syringe equipped with a needle, ensuring the removal of any bubbles to achieve a uniform electrospun nanofiber structure. This meticulous preparation process was critical for producing high-quality nanofibers with consistent properties for subsequent applications. The needle was attached to one end of the power source, and the syringe was placed onto the syringe pump. The collecting mandrel was attached to the opposite end of the power supply after being wrapped in freshly cleaned aluminum foil. The distance between the collecting mandrel and the needle was twelve centimeters. The nanofibers were electrospun at 10.66 kV, and the flow rate was set to 1 mL h^−1^. The fiber morphology was systematically optimized by modulating three critical parameters: (1) PAN and Bio-Ag NPs@PAN concentrations (8–14% w/w), (2) flow rate (0.2–2 mL h^−1^), and (3) needle gauge (19–22G). SEM analysis (Fig. 3a, c and e) quantitatively confirmed that each parameter dominantly controls distinct structural features, as summarized in Table 1.

**Table 1:** Parameter optimization for PAN and Bio-Ag NPs@PAN fiber morphology^a^

Parameter	Tested Range	Key morphological impact	Optimal value
PAN and Bio-Ag NPs@PAN concentrations	8–14% w/w	↑Conc.: fiber diameter↑; bead formation↓; ribbon like fibers at >12%	10% w/w
Flow rate	0.2–2 mL h^−1^	↑Flow: diameter↑; beads↑ (>1.5 mL h^−1^); porosity↑	1 mL h^−1^
Needle gauge	19–22G (*Ø* 0.4–0.2 mm)	↑Gauge (smaller *Ø*): diameter↓; fiber uniformity↑; jet stability↓ at >22G	21G (*Ø* 0.31 mm)

a The structural framework of the electrospinning setup is demonstrated in Scheme 1c.

### 2.6. Characterization of the Bio-Ag NPs, PAN, and Bio-Ag NPs@PAN

A comprehensive set of characterization techniques were employed to analyze the synthesized nanoparticles and nanofibers, including using a UV-vis spectrophotometer (Yoke Instrument, Shanghai, China) to scan absorption spectra across 200–800 nm at 1 nm resolution. As reported in previous studies, the surface plasmon resonance of Ag NPs results in a yellowish-brown coloration in both solvent and aqueous environments.^68^ In this study, the addition of *S. guineense* extracts to the aqueous silver nitrate solution induced a color transition from pale yellow to yellowish brown, then reddish brown, and finally colloidal brown, indicating the successful formation of Ag NPs. This color change aligns with observations in other studies,^69–71^ confirming the completion of the reaction between the both extracts and AgNO_3_. Functional groups were characterized using Fourier-transform infrared spectroscopy (FTIR; Bruker INVENIO S, UK). FTIR spectroscopy was used to identify the biomolecules present in the extracts, and the synthesized nanoparticles. The FTIR analysis confirmed the presence of various phytochemicals in the plant extracts, including anthraquinone glycosides, tannins, phenolic compounds, alkaloids, and saponins.^33^ X-ray diffraction (XRD) patterns were acquired using a Rigaku Ultima IV diffractometer (Tokyo, Japan) with Cu Kα radiation (*λ* = 1.5406 Å) at 40 kV/40 mA, scanning 2*θ* angles from 5° to 80°. XRD analysis revealed the diffraction patterns and lattice planes corresponding to the face-centered cubic (FCC) structure of AgNPs, which matched the data reported in ICDD file No. 00-004-7383.^72^ Surface morphological analysis was conducted using an SU-8010 scanning electron microscope (SEM), and energy dispersive X-ray spectroscopy (EDX) was used to determine the elemental composition of samples (SEM-EDX; Hitachi High-Technologies Co., Ltd, Tokyo, Japan). SEM analysis was conducted after the initial centrifugation of the Ag NP-containing colloidal solutions at 4000 rpm for 15 minutes. The separated pellets were collected, while the supernatants underwent a second centrifugation at 10 000 rpm for 20 minutes to ensure complete purification. The resulting supernatants were discarded, and the purified pellets were resuspended in 0.1 mL of deionized water. The mixture was then thoroughly homogenized, applied onto a glass coverslip, and air-dried for SEM analysis.^73^ Transmission Electron Microscopy (TEM) (HT7700 High-Technologies Co., Ltd, Tokyo, Japan) was used to reveal nanoscale structure, morphology, crystallography, and composition. These characterizations collectively validated the successful synthesis and structural integrity of the Bio-Ag NPs and Bio-Ag NPs@PAN, highlighting their potential for various biomedical applications.

### 2.7. Antibacterial analysis

The plates containing nutritious agar medium were swabbed using the microbial cultures. The discs were immersed in the following solutions: a 1 mM solution of silver nanoparticles produced by plant-mediated synthesis; plant leaf extracts; and double-distilled water as a negative control. The prepared plates were subsequently incubated at 37 °C for 24 hours to assess antibacterial activity.^73^ The results indicated that *S. guineense*-derived Ag NPs exhibited stronger antibacterial activity against Gram-positive bacteria compared to Gram-negative bacteria. This difference is likely due to structural variations in bacterial cell walls, where the thicker peptidoglycan layer in Gram-positive bacteria may enhance Ag NP interaction. Further, the bioactive compounds present in the plant extracts, which act as capping and stabilizing agents on the nanoparticle surface, may contribute to their antibacterial properties.^74^

### 2.8. Antioxidant activity assessment using DPPH assay

The antioxidant potential of the extracts and Bio-Ag NPs was evaluated using the stable DPPH (2,2-diphenyl-1-picrylhydrazyl) radical assay. The absorbance was measured at 517 nm, and the DPPH radical scavenging activity was calculated using the following equation:1
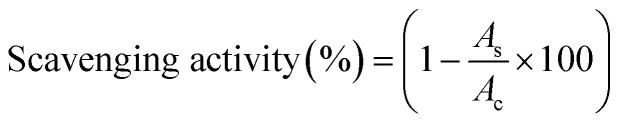
where ‘*A*_c_’ denotes the absorbance of the control reaction and ‘*A*_s_’ denotes the sample's absorbance. The term “IC_50_” refers to the antioxidant activity of both the extracts and Bio-Ag NPs, representing the concentration required to inhibit 50% of DPPH radical activity. Specifically, the IC_50_ value indicates the concentration (mg mL^−1^) of the dry material necessary to achieve 50% inhibition of DPPH radical production. Each IC_50_ value was calculated using the regression equation derived from the experimental data.^75^

### 2.9. Preparation of *S. guineense* leaf and stem bark extracts, Bio-Ag NPs(L), and Bio-Ag NPs(B) for DPPH analysis

To prepare the samples for the DPPH (2,2-diphenyl-1-picrylhydrazyl) assay, 5 g of leaf and stem bark samples was placed into 100 mL beakers, and 50 mL of 99% methanol was mixed with each. The mixtures were then placed in a shaking water bath set at 100 rpm and 29.2 °C for 3 hours to facilitate extraction. Concurrently, a DPPH solution was prepared by dissolving 4 mg of DPPH in 100 mL of 99% methanol. This solution was sealed with aluminum foil and stored in a dark, cool environment to prevent degradation. After incubation, the extracts were centrifuged at 8000 rpm for 15 minutes, and the supernatant was vacuum-filtered to remove any particulate matter. The filtered extracts were then aliquoted into volumes of 1, 2, 3, 4, 5, 6, 7, and 8 mL, with each aliquot diluted with an equal volume of 99% methanol. Next, 1 mL of each diluted extract was mixed with 3 mL of the prepared DPPH solution, followed by the addition of 10 mL of 99% methanol. The mixtures were incubated in the dark for 30 minutes to allow the reaction to proceed. Final concentrations of 40, 50, 60, 70, and 80 mg mL^−1^ were achieved for the assay. Ascorbic acid and methanol were used as positive and negative controls, respectively. The DPPH assay was performed by measuring the absorbance of the solutions at 517 nm using a spectrophotometer. This method allowed for the evaluation of the antioxidant activity of the extracts based on their ability to scavenge free radicals, as indicated by a reduction in absorbance compared to the control.

### 2.10. *In vivo* diabetic wound healing

#### 2.10.1. *In vivo* experimental descriptions

Sprague-Dawley white rats (SD) were purchased from Sibeifu (Beijing) Biotechnology Co., Ltd (Production License No.: SCXK (Beijing) 2024-0001). The animals were kept in the SPF animal laboratory at the Clinical Research Institute of China-Japan Friendship Hospital under controlled conditions, maintaining a constant temperature of 22–24 °C and humidity of 55 ± 5%, with artificial light and darkness for 12 hours, and free access to drinking water and food. Male SD rats (7–8 weeks old, weighing 230 g) were first acclimated for seven days. Following a 12-hour fasting period, the SD rats were intraperitoneally injected with streptozotocin (STZ) at a dosage of 60 mg kg^−1^ (GC301002-100 mg, Servicebio, Wuhan, China). Fasting blood glucose levels were subsequently measured by collecting blood from the rat tail vein. Diabetes was confirmed in rats when blood glucose levels exceeded 250 mg dL^−1^ for three consecutive days, successfully establishing the diabetic rat model.^76^ A 10 mm diameter incision wound was made between the two scapulae on the back of diabetic rats under respiratory anesthesia, and a full-thickness skin defect model of diabetic rats was established.^77^ The rats were randomly assigned to four groups: the control group, the PAN group, the Bio-Ag NPs(L)@PAN group, and the Bio-Ag NPs(B)@PAN group, with four rats in each group. Wound healing progression was observed and documented through photography on days 0, 3, 7, 11, and 14.^78^ After 14 days, the rats were euthanized *via* intraperitoneal injection of 6% pentobarbital sodium (3 mL kg^−1^). The full-thickness skin tissue from the surgical site was excised and fixed in 4% paraformaldehyde for pathological examination. This study was approved by the Animal Ethics Committee of China-Japan Friendship Hospital (Approval Number: ZRDWLL240089), ensuring compliance with ethical standards for animal research.

#### 2.10.2. Evaluation of diabetic wound healing progress

The extent of diabetic wound healing was assessed by measuring the wound area at specific time intervals. Wound images were captured on 0, 3, 7, 11, and 14 days using a digital camera. The wound area was analyzed using ImageJ software to quantify the percentage of wound closure over time. The wound contraction rate was calculated using the following equation:2
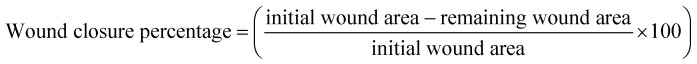


Histological analyses were conducted on the collected wound tissues, which were fixed in 4% paraformaldehyde and embedded in paraffin. Sections were stained with hematoxylin and eosin (H&E) and Masson's trichrome to evaluate re-epithelialization, collagen deposition, and inflammatory cell infiltration. These histological observations provided insights into the healing process and tissue regeneration across post-treatment.

### 2.11. Statistical analysis

The data were statistically analyzed using SPSS 21.0, while graphing and visualization were performed using Origin 2024 SR1 (version 10.10.178) and BioRender software. Descriptive statistics, including mean percentages and standard deviation (SD), were calculated for each variable to summarize the data. For comparative analysis, a one-way analysis of variance (ANOVA) was conducted, followed by Tukey's post hoc test to identify specific differences between groups. A *p*-value of less than 0.05 (*p* < 0.05) was considered statistically significant, ensuring the reliability and validity of the findings. This rigorous statistical approach provided a robust framework for interpreting the experimental results.

## 3 Results and discussion

### 3.1. Characterization of the chemical structure

**Fig. 1:**
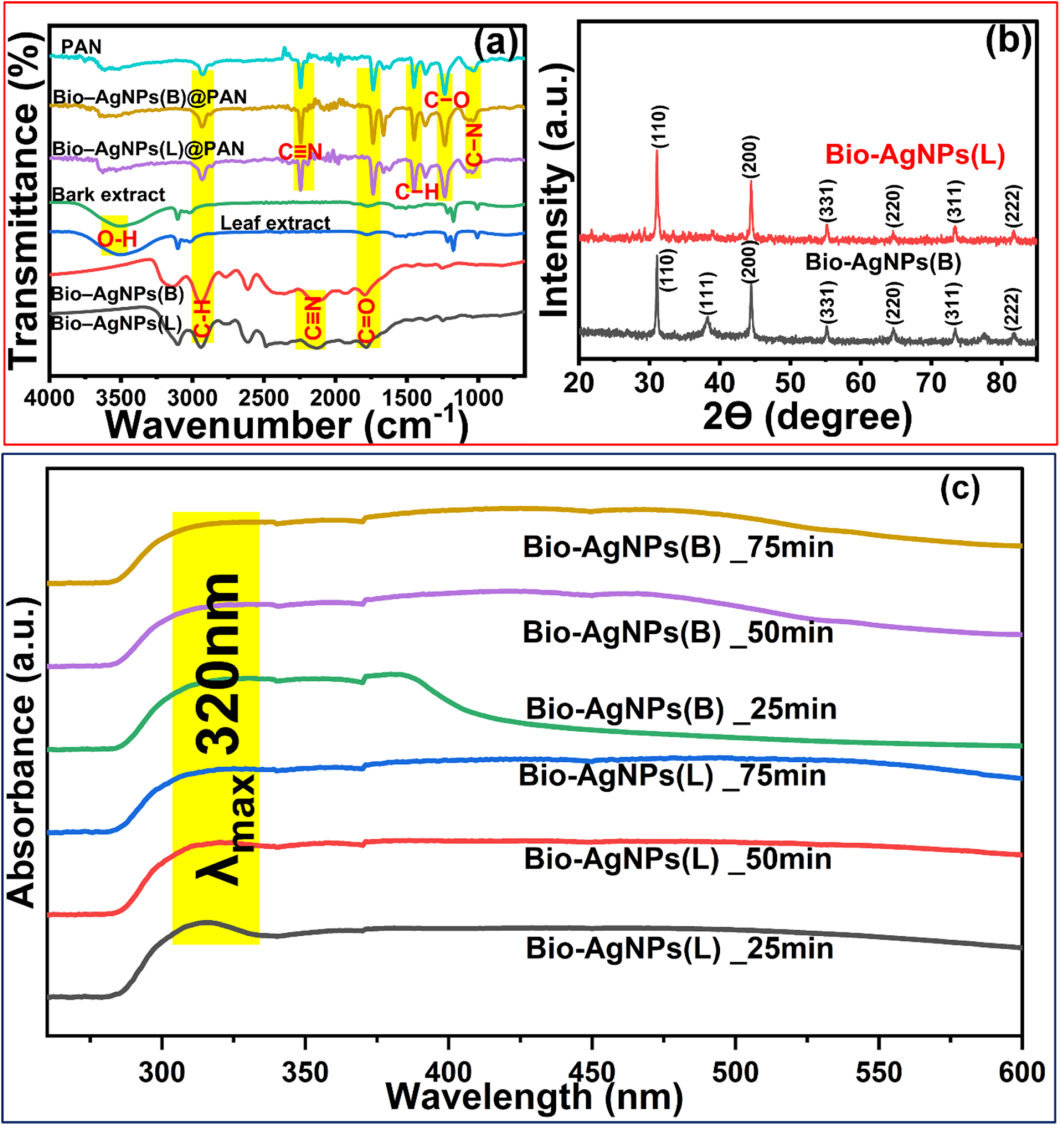
(a) FTIR spectra, (b) XRD patterns, and (c) UV-visible spectra of Bio-Ag NPs synthesized from *S. guineense* leaf extract (Bio-Ag NPs(L)) and stem bark extract (Bio-Ag NPs(B)).

Fig. 1a presents the FTIR spectra of *S. guineense* leaf and bark extracts, revealing a broad peak in the range of 3598–3425 cm^−1^, which corresponds to –OH stretching vibrations, indicating the presence of hydroxyl groups. Peaks observed at 3100 cm^−1^ are attributed to C–H sp^2^ hybrid bonds, while the absence of peaks in the triple bond region confirms the lack of –C≡N– groups in both extracts. Following the biosynthesis of Bio-Ag NPs(L) and Bio-Ag NPs(B), a characteristic peak emerged at 2954 cm^−1^, signifying sp^3^ hybrid C–H stretching vibrations. Additionally, a peak at approximately 2240 cm^−1^ in both Bio-Ag NPs(L) and Bio-Ag NPs(B) confirmed the presence of –C≡N– triple bonds. A peak at 1735 cm^−1^ was observed, corresponding to the <svg xmlns="http://www.w3.org/2000/svg" version="1.0" width="10.400000pt" height="16.000000pt" viewBox="0 0 10.400000 16.000000" preserveAspectRatio="xMidYMid meet"><metadata> Created by potrace 1.16, written by Peter Selinger 2001-2019 </metadata><g transform="translate(1.000000,15.000000) scale(0.011667,-0.011667)" fill="currentColor" stroke="none"><path d="M80 1160 l0 -40 40 0 40 0 0 -40 0 -40 40 0 40 0 0 -40 0 -40 40 0 40 0 0 -40 0 -40 40 0 40 0 0 -40 0 -40 40 0 40 0 0 -40 0 -40 40 0 40 0 0 -40 0 -40 40 0 40 0 0 80 0 80 -40 0 -40 0 0 40 0 40 -40 0 -40 0 0 40 0 40 -40 0 -40 0 0 40 0 40 -40 0 -40 0 0 40 0 40 -40 0 -40 0 0 40 0 40 -80 0 -80 0 0 -40z M560 520 l0 -40 -40 0 -40 0 0 -40 0 -40 -40 0 -40 0 0 -40 0 -40 -40 0 -40 0 0 -40 0 -40 -40 0 -40 0 0 -40 0 -40 -40 0 -40 0 0 -40 0 -40 -40 0 -40 0 0 -40 0 -40 80 0 80 0 0 40 0 40 40 0 40 0 0 40 0 40 40 0 40 0 0 40 0 40 40 0 40 0 0 40 0 40 40 0 40 0 0 40 0 40 40 0 40 0 0 80 0 80 -40 0 -40 0 0 -40z"/></g></svg> C=O stretching of carbonyl groups, further supporting the structural modifications induced by nanoparticle synthesis. The FTIR spectra of PAN, Bio-Ag NPs(L)@PAN, and Bio-Ag NPs(B)@PAN nanofibers also displayed distinctive peaks. The peak at 2954 cm^−1^ confirmed the presence of sp^3^ hybrid C–H stretching vibrations, while the peak at 1735 cm^−1^ corresponded to the C=O stretching of carbonyl groups. The moderate sharp peak at 1049 cm^−1^ was attributed to the –C–O stretching of phenolic compounds. The subtle peak at around 883 cm^−1^ indicated the presence of an Ag–O bond, arising from the bending vibrations of Ag–O–H bonds. The peak at 2240 cm^−1^ in the nanofibers confirmed the presence of –C≡N– triple bonds. Additional identified peaks included the amine –C−N stretching vibrational band at approximately 1060 cm^−1^, –C=C– stretching at 1662 cm^−1^, and –C–H scissoring at 1450 cm^−1^.

FTIR analysis further confirmed that *S. guineense* leaf and stem bark extracts contain several essential phytochemicals, such as flavonoids, phenolic compounds, and alkaloids, which play a crucial role in the biosynthesis of Bio-Ag NPs. These findings highlight the functional groups and structural characteristics of the extracts and synthesized nanoparticles, providing valuable insights into their chemical composition and potential applications.

Fig. 1b displays the XRD patterns of Bio-Ag NPs(L) and Bio-Ag NPs(B), confirming their crystalline nature. The diffraction peaks corresponding to the face-centered cubic (FCC) lattice planes of Bio-Ag NPs(L) appear at 2*θ* = 31.06° (110), 44.3° (200), 54.6° (331), 64.5° (220), 77.5° (311), and 81.6° (222). Similarly, the diffraction peaks for Bio-Ag NPs(B) are observed at 31.06° (110), 38.2° (111), 44.3° (200), 54.6° (331), 64.5° (220), 77.50° (311), and 81.6° (222) which was consistent with previous studies.^50,79,80^ The presence of these distinct peaks confirms the successful biosynthesis of crystalline Bio-Ag NPs using *S. guineense* plant extracts. The crystalline size of the Bio-Ag NPs corresponding to each lattice plane was determined using Debye–Scherrer's equation.^79,81^3
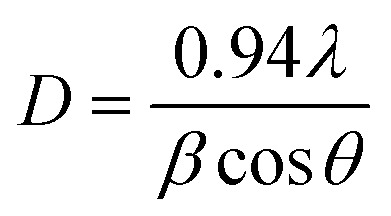


The average crystalline size of the Bio-Ag NPs, calculated across all lattice planes, was determined to be 17.35 nm for Bio-Ag NPs(L) and 17.42 nm for Bio-Ag NPs(B). The detailed crystalline size corresponding to each diffraction plane is provided in Tables S1 and S2 (SI).

Fig. 1c displays the UV-vis absorbance spectrum of Bio-Ag NPs, recorded at a *λ*_max_ of 320 nm after 25 minutes. This absorption band is primarily attributed to the surface plasmon resonance (SPR) of Bio-Ag NPs, a characteristic feature of silver nanoparticles. Similar absorbance peaks at a *λ*_max_ of 320 nm were observed at 50 and 75 minutes, demonstrating consistent and stable nanoparticle formation over time. While phyto-synthesized Ag NPs typically exhibit an SPR peak at 400–450 nm, a blue shift to 320–380 nm signals dense phytochemical capping altering the NP's surface environment and/or incomplete reduction of silver precursors. Moreover, the tiny size of Ag NPs causes a shorter wavelength to appear in the UV-vis spectra; in contrast, a bigger size causes a longer wavelength.^66,82^ The reduction of silver ions (Ag^+^) to silver atoms (Ag^0^) is facilitated by biomolecules present in the plant leaf and stem bark extracts, which act as both reducing and stabilizing agents. This process initiates the nucleation of small silver atom clusters, which eventually aggregate to form nanoparticles. Polyphenolic compounds, in particular, play a key role in reducing silver ions to silver atoms, which then coalesce into nanoparticles. Over time, the silver atoms undergo gradual oxidation, leading to the formation of Ag_2_O (silver oxide), further confirming the dynamic transformation and stabilization of Bio-Ag NPs. Ag_2_O, a semiconductor with a bandgap of 1.2 eV,^83^ generates electron–hole (e^−^–h^+^) pairs under UV-visible irradiation. Concurrently, phytochemicals in *S. guineense* extracts (*e.g.*, phenolics, aldehydes) act as electron donors, reducing Ag^+^ in Ag_2_O to Ag^0^. This effect drives the photoreduction of Ag^+^ to metallic Ag^0^ through the two synergistic electrochemistry equations; the first pathway is direct photocatalytic reduction:^84–86^

4 Ag_2_O + *hν* → 2Ag^+^ + 2e^−^ + ½O_2_ (photoexcitation)

5 Ag^+^ + e^−^ → Ag^0^ (reduction by photoelectrons)

The second pathway is biomolecule-mediated reduction, which was the residual of organic compounds (such as R–CHO aldehydes) in the bio-capping layer reducing Ag_2_O:

6 Ag_2_O + 2H^+^ + R–CHO → 2Ag^0^ + H_2_O + R–COOH

The combined action reforms Bio-Ag NPs, while oxidized phytochemicals like R–COOH enhance colloidal stability *via* surface coordination.^87,88^ This demonstrated that Bio-Ag NPs were successfully synthesized.

**Fig. 2:**
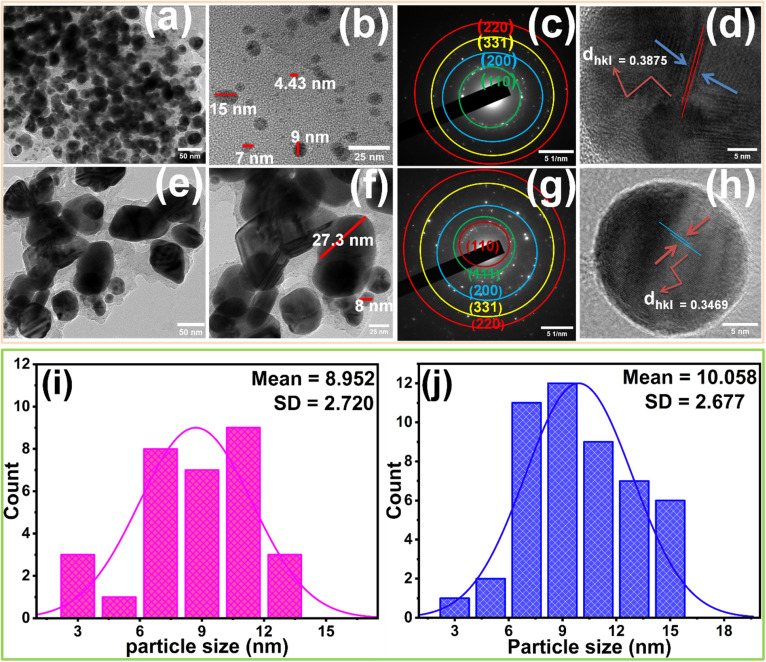
(a and b) TEM images of Bio-Ag NPs(L); (c and d) SAED patterns of Bio-Ag NPs(L); (e and f) TEM images of Bio-Ag NPs(B); (g and h) SAED patterns of Bio-Ag NPs(B), TEM analysis of Bio-Ag NPs showing particle size distribution for (i) Bio-Ag NPs(L) and (j) Bio-Ag NPs(B).

The TEM micrographs of Bio-Ag NPs(L) and Bio-Ag NPs(B) are shown in Fig. 2a, b, e and f revealing their finely grained structure with diverse morphologies, including nearly spherical, triangular, hexagonal, prismatic, and cylindrical shapes. The selected area electron diffraction (SAED) patterns in Fig. 2c, d, g and h exhibit four distinct diffraction spots for Bio-Ag NPs(L) and five for Bio-Ag NPs(B), corresponding to the crystalline planes of (110), (200), (331), and (220) for Bio-Ag NPs(L), and (110), (111), (200), (331), and (220) for Bio-Ag NPs(B). High-resolution TEM (HRTEM) images confirm lattice fringes with *d*-spacing values of 0.3875 nm for Bio-Ag NPs(L) and 0.3469 nm for Bio-Ag NPs(B), indicating their crystalline nature. Fig. 2i and j depict the particle size distribution histograms of Bio-Ag NPs(L) and Bio-Ag NPs(B). The relatively uniform size distribution, ranging from 4.43 nm to 15 nm for Bio-Ag NPs(L) and 8 nm to 27.3 nm for Bio-Ag NPs(B), indicates that the biosynthesis process successfully produced monodispersed nanoparticles. A narrower size distribution enhances nanoparticle stability, as uniform particles are less prone to aggregation over time. Additionally, size distribution plays a crucial role in determining the surface-area-to-volume ratio, which directly influences the antibacterial efficacy of the nanoparticles. Smaller particles provide a greater number of active sites, thereby enhancing antimicrobial activity. These characteristics highlight the potential of Bio-Ag NPs for different applications, particularly in antibacterial treatments and wound healing.

**Fig. 3:**
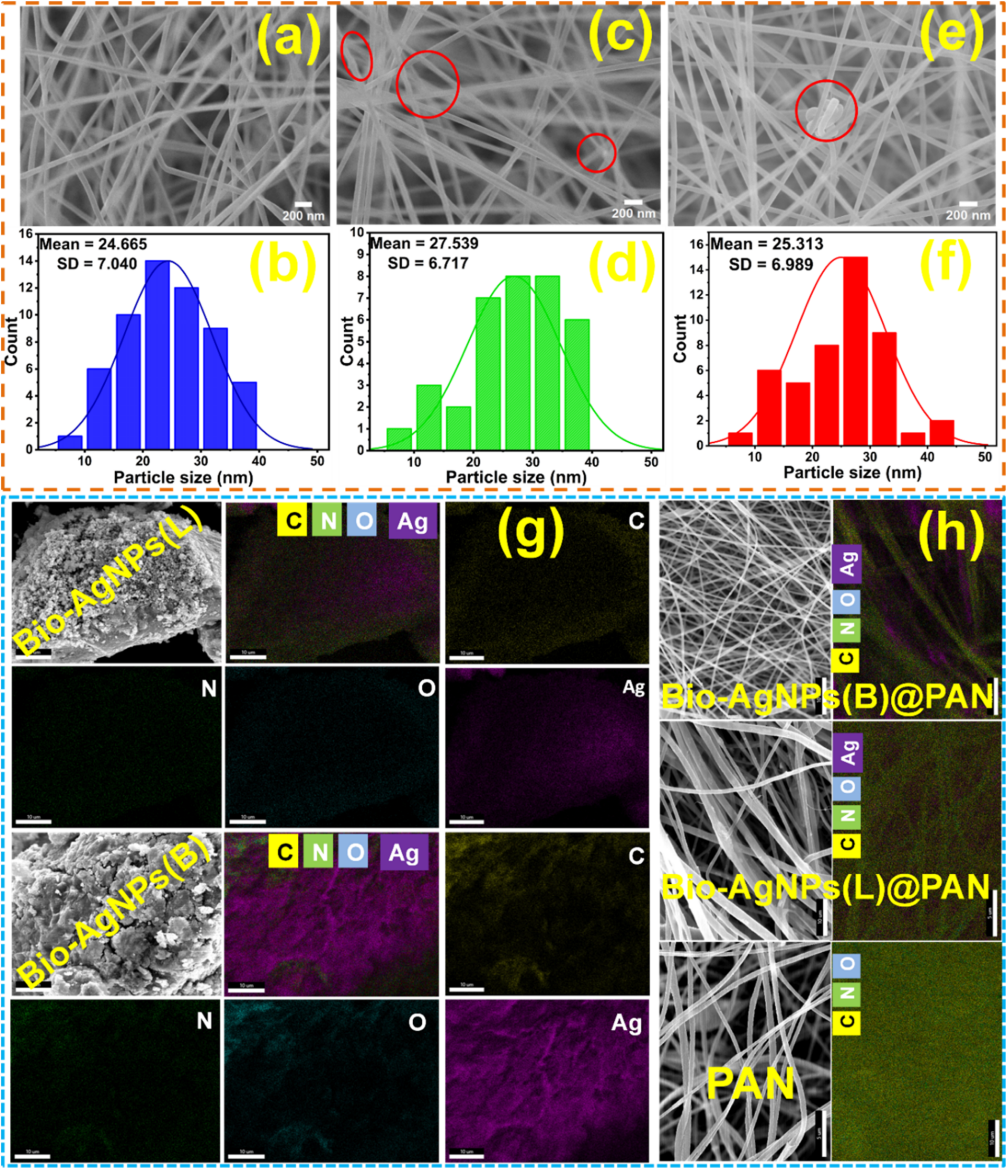
SEM images of PAN (a) and its particle size distribution (b); Bio-Ag NPs(L)@PAN (c) and its particle size distribution (d); and Bio-Ag NPs(B)@PAN (e) and its particle size distribution (f); EDX spectra of Bio-Ag NPs (g), PAN alone and Bio-Ag NPs@PAN (h).

SEM analysis was conducted to examine the morphology of PAN nanofibers and the structural modifications resulting from the incorporation of *S. guineense* leaf and stem bark extracts containing Bio-Ag NPs. Fig. 3a, c, and e present SEM images of PAN alone, Bio-Ag NPs(L)@PAN, and Bio-Ag NPs(B)@PAN, respectively, offering detailed insights into the nanofiber characteristics. The SEM images reveal that the polymer nanofibers exhibit a smooth, uniform, and cylindrical morphology, indicative of a well-organized structure. The incorporation of *S. guineense* leaf and stem bark extracts not only introduced bioactive compounds but also served as natural reducing and capping agents, facilitating the formation of a robust and well-structured nanofiber network. These bioactive compounds played a crucial role in ensuring the stability, homogeneity, and enhanced structural integrity of the synthesized Bio-Ag NPs and nanofibers. The results demonstrate that the integration of *S. guineense* extracts containing Bio-Ag NPs with PAN significantly enhances the properties of the nanofibers, making them highly suitable for advanced applications in biomedical fields.

EDX analysis was performed to confirm the successful synthesis of Bio-Ag NPs(L) and Bio-Ag NPs(B) derived from *S. guineense* leaf and stem bark extracts. For Bio-Ag NPs(L), the weight percentages of C, N, O, and Ag were determined to be 65.24%, 19.79%, 10.26%, and 4.71%, respectively. Similarly, the EDX analysis of Bio-Ag NPs(B) revealed weight percentages of C, N, O, and Ag of 59.00%, 17.41%, 13.67%, and 9.92%, respectively, as illustrated in Fig. 3g and S1. Additionally, the EDX spectra of Bio-Ag NPs(L)@PAN and Bio-Ag NPs(B)@PAN confirmed the presence of C, N, O, and Ag, with significant elemental peaks observed in the EDX elemental maps, as highlighted in Fig. 3h and S1. For Bio-Ag NPs(L)@PAN, the weight percentages of C, N, O, and Ag were 63.85%, 20.25%, 11.45%, and 4.44%, respectively. In contrast, Bio-Ag NPs(B)@PAN exhibited weight percentages of C, N, O, and Ag of 60.05%, 20.26%, 10.41%, and 9.29%, respectively.

Furthermore, the EDX spectrum of pure PAN confirmed the presence of C, N, and O, with weight percentages of 73.84%, 19.23%, and 6.93%, respectively. These results validate the successful incorporation of Bio-Ag NPs into the PAN nanofibers and highlight the presence of Bio-Ag NPs in the nanocomposite materials. The EDX analysis provides strong evidence for the synthesis and integration of Bio-Ag NPs.

### 3.2. Antibacterial test results

**Fig. 4:**
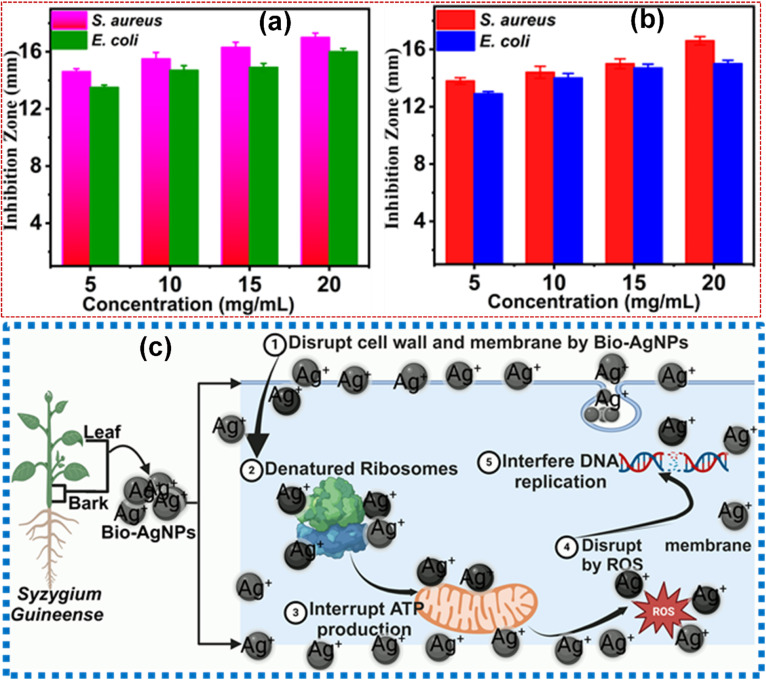
Antibacterial activity of (a) Bio-Ag NPs(L) and (b) Bio-Ag NPs(B). Data were analyzed using one-way ANOVA followed by Tukey's multiple comparisons test and are presented as mean ± SD, with statistical significance set at *P* < 0.05. (c) Schematic illustration depicting the antibacterial mechanism of bio-synthesized silver nanoparticles (Bio-Ag NPs).

The antibacterial properties of Bio-Ag NPs were evaluated against two bacterial strains: Gram-positive (*S. aureus*) and Gram-negative (*E. coli*). The results demonstrated that Bio-Ag NPs exhibited significant antibacterial activity, with inhibition zones of 17.0 ± 0.310 mm for *S. aureus* and 16.3 ± 0.290 mm for *E. coli*. Due to structural differences in bacterial cell walls, Bio-Ag NPs exhibited a higher antibacterial effect against Gram-positive bacteria compared to Gram-negative bacteria. In this study, leaf and stem bark extracts of *S. guineense*, along with Bio-Ag NPs, were tested at a concentration of 20 mg mL^−1^, resulting in inhibition zones ranging from 10 to 17 mm. The highest inhibition zone of 17 mm was observed against *S. aureus*, followed by 16.3 mm against *E. coli*. Lower concentrations (5–15 mg mL^−1^) of the leaf and stem bark extracts, as well as Bio-Ag NPs, also demonstrated significant antibacterial activity against both bacterial strains. These findings highlight that antibacterial efficacy is influenced by both concentration and the type of bacterial strain. At higher concentrations, *S. aureus* exhibited greater susceptibility compared to *E. coli*, indicating a stronger inhibitory effect on the Gram-positive strain. The antibacterial assay, as illustrated in Fig. 4a and b and detailed in **Tables S3** and **S4**, demonstrated that *S. guineense* leaf and stem bark extracts, along with Bio-Ag NPs(L) and Bio-Ag NPs(B), exhibited potent and progressively increasing antibacterial activity at different concentrations. This concentration-dependent activity underscores the potential of these extracts and Bio-Ag NPs as effective antibacterial agents, particularly against Gram-positive bacteria more like *S. aureus* but also Gram-negative bacteria like *E. coli*. These results further validate the therapeutic potential of *S. guineense*-derived Bio-Ag NPs for applications in combating bacterial infections.

#### 3.2.1. Antibacterial mechanisms of Bio-Ag NPs

The hypothesized mechanism of Ag NPs' antibacterial action involves their accumulation in the bacterial cell wall, disrupting the lipid bilayer and compromising membrane integrity.^50^ Ag NPs or the released silver ions (Ag^+^) can penetrate bacterial cells, where they interact with phosphorus and sulfur-containing biomolecules, inhibiting protein synthesis and halting DNA replication. The antibacterial activity of Ag NPs is significantly influenced by the surface area of the nanomaterial;^89^ that with a larger surface area releases higher amounts of Ag^+^ ions, enhancing their antibacterial efficiency. In contrast, Ag NPs with a smaller surface area exhibit limited Ag^+^ ion release, leading to weaker antibacterial activity.^90^ Ag NPs have unique physical and chemical characteristics that allow them to interact with bacterial cells in multifaceted ways.^91^ They possess a broad spectrum of antibacterial properties due to their ability to permeate bacterial cell walls. They alter the structure of cell membranes, which can lead to cell death.^92^ The efficacy of these nanoparticles is attributed to their nanoscale size and large surface area-to-volume ratio, allowing for enhanced interaction with microbial cells. The exact mechanisms behind the antibacterial action of Ag NPs are complex. However, it has been suggested that Ag NPs can continuously release silver ions, which play a crucial role in inhibiting bacterial growth. The silver ions adhere to the bacterial cell wall and cytoplasmic membrane due to electrostatic interactions, thereby increasing membrane permeability and leading to cell envelope disruption.^93^ Silver ions can also penetrate the bacterial cells, where they deactivate respiratory enzymes, generate reactive oxygen species (ROS), and interfere with adenosine triphosphate (ATP) production. This disruption in energy production can ultimately result in cell death, as this process is vital for cellular functions and replication.^91,94^ The mechanism of antibacterial inhibition is shown in Fig. 4c. This study provides a consolidated review of published studies on the antibacterial activity of silver nanoparticles (Ag NPs) synthesized using different plant extracts, enabling a comparative assessment of their efficacy, which are tabulated in Table 2.

**Table 2:** Summary of literature reviews on the antibacterial properties of Ag NPs synthesized using various plant extracts for comparative analysis

S. no.	Name of plants	Inhibition zone (mm)	Bacterial strains	References
1	*Bergenia ciliata*	8.5	*S. aureus*	95
2	Balloon flower plants	12	*E. coli*	96
3	*Aloe fleurentiniorum*	12, 14.5	*E. coli*, *S. aureus*	97
4	*Chenopodium murale*	12.7	*S. aureus*	98
**5**	***Syzygium guineense***	**17, 16.6, 15.5, 16.3, 16, 15**	***S. aureus*, *E. coli***	**This study**
6	*Ocimum Sanctum* (Tulsi)	14	*E. coli*	99
7	Banana peel	17, 16	*E. coli*, *S. aureus*	100

### 3.3. Antioxidant activity in DPPH assay

**Fig. 5:**
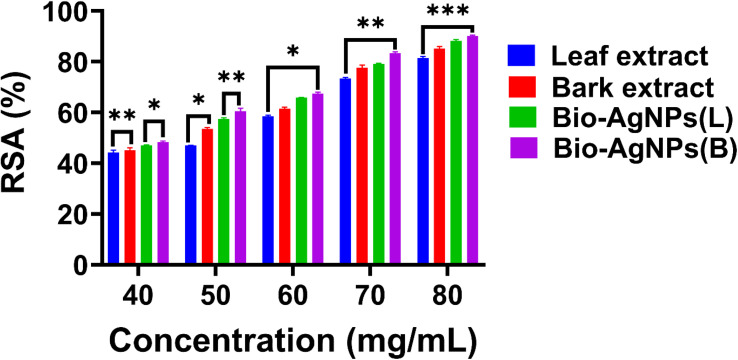
DPPH assay findings.

The synthesized Bio-Ag NPs(L) and Bio-Ag NPs(B) exhibited significantly enhanced antioxidant activity compared to the raw plant extracts. This improvement is attributed to the nanoparticles' small size, large surface area, and unique physicochemical properties. The inherent antioxidant properties of Bio-Ag NPs(L) and Bio-Ag NPs(B) enable them to effectively scavenge free radicals and promote the bioactivity of plant-derived molecules, especially Bio-Ag NPs. The results revealed a scavenging activity of 48.53% at the lowest concentration (40 mg mL^−1^) with an absorbance of 0.603 and 90.60% at the highest concentration (80 mg mL^−1^) with an absorbance of 0.202. These findings further validate the study, showing that as concentration increased, absorbance decreased while scavenging activity enhanced, eventually reaching its maximum threshold. The regression (*R*^2^) values for the leaf and stem bark extracts were 0.931 and 0.9612, respectively, while Bio-Ag NPs(L) and Bio-Ag NPs(B) exhibited even higher *R*^2^ values of 0.9976 and 0.9921, respectively, indicating a strong correlation with antioxidant activity. As shown in **Tables S5** and **S6**, the IC_50_ values of the leaf and stem bark extracts were 9.23 and 36.01 at the lowest concentration (40 mg mL^−1^) and 47.293 and 72.36 at the highest concentration (80 mg mL^−1^). In contrast, the IC_50_ values of Bio-Ag NPs(L) and Bio-Ag NPs(B) were 39.29 and 41.08 at 40 mg mL^−1^, and 75.37 and 81.66 at 80 mg mL^−1^, respectively. The antioxidant scavenging efficiency in the DPPH assay is illustrated in Fig. 5, further validating the superior antioxidant potential of Bio-Ag NPs(L) and Bio-Ag NPs(B) compared to the raw extracts. This highlights the enhanced bioactivity of the Bio-Ag NPs, making them promising candidates for applications in oxidative stress-related therapies and other biomedical fields.

The antioxidant mechanism of *S. guineense* plant extracts, Bio-Ag NPs(L) and Bio-Ag NPs(B), is attributed to their high surface-to-volume ratio, which enhances their interaction with free radicals, leading to effective radical scavenging. The bioactive compounds in *S. guineense* extracts, including polyphenols, flavonoids, and alkaloids act synergistically with Bio-Ag NPs to enhance their antioxidant efficacy. Moreover, silver ions (Ag^+^) released from the Bio-Ag NPs further contribute to antioxidant activity by neutralizing free radicals. Antioxidants play a crucial role in preventing oxidative damage by inhibiting reactive oxygen species (ROS), which can impair cellular function.^101,102^ These findings underscored the potential of Bio-Ag NPs as effective antioxidant agents with promising therapeutic applications in diabetic wound healing.

### 3.4. Characterization of diabetic wound healing and closure

**Fig. 6:**
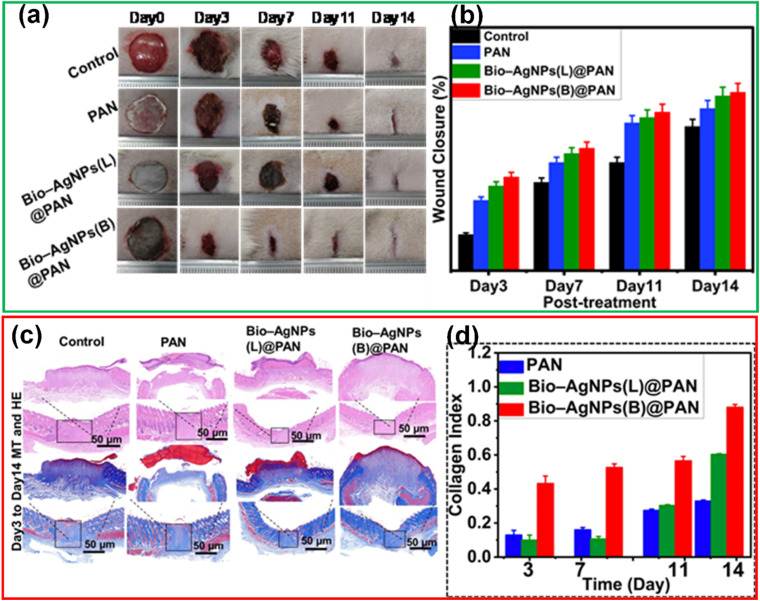
*In vivo* diabetic wound healing process: (a) representative images showcasing wound healing progression over time. (b) Quantitative analysis of wound closure (%) on days 3, 7, 11, and 14 post-treatments. (c) Histological evaluation of wound sections using Masson's Trichrome (MT) and Haematoxylin & Eosin (HE) staining, highlighting tissue morphology and collagen distribution. (d) Collagen deposition analysis post-treatment. Data are expressed as mean ± SD (*n* = 4), with statistical significance set at *p* < 0.05.

According to the wound closure (%) data in Fig. 6a and b, all treatment groups exhibited progressive healing, with the injured skin showing significant reepithelialization from day 3 to day 14. Among the nanofiber-treated groups, Bio-Ag NPs(L)@PAN and Bio-Ag NPs(B)@PAN exhibited the highest wound closure percentage on day 3, consistently maintaining this trend through days 7, 11, and 14. Although wound closure was observed in both the PAN nanofiber-treated and control groups, it was significantly lower compared to the groups treated with Bio-Ag NPs(L)@PAN and Bio-Ag NPs(B)@PAN. As the treatment period advanced, the percentage of wound closure steadily increased across all groups. Bio-Ag NPs(B)@PAN exhibited the highest wound closure percentages on days 3 (52%), 7 (68%), 11 (88%), and 14 (99%), significantly outperforming the control group, which achieved 20%, 49%, 60%, and 71% wound closure, respectively (*p* < 0.05). On the fourteenth day post-incision, the wounds treated with Bio-Ag NPs(B)@PAN (99%) and Bio-Ag NPs(L)@PAN (97%) were nearly fully healed, aligning with findings from previous literature.^53^ At the 0.05 significance level, the mean ± SD values for wound closure were significantly different post-treatment. The Bio-Ag NPs(L)@PAN and Bio-Ag NPs(B)@PAN groups exhibited superior wound healing, with complete reepithelialization, near-total wound closure, and an almost intact arrangement of collagen fibers in the dermis, in contrast to the control group.

### 3.5. Histological analysis of diabetic wound tissue

Hematoxylin and Eosin (HE) staining and Masson's Trichrome (MT) staining are widely used in pathology and biomedical research to analyze tissue sections. HE staining assesses tissue architecture, cellular morphology, and overall organ structure, while MT staining specifically highlights collagen fibers. The standard deviation (SD) quantifies variability in measurements, where a lower SD indicates high consistency and minimal variation, while a higher SD reflects greater variability. The mean area percentage of MT staining measures the proportion of collagen fibers relative to the total tissue area. Throughout the study period, the mean collagen area percentage for Bio-Ag NPs(L)@PAN and Bio-Ag NPs(B)@PAN confirmed that collagen levels significantly occupied the tissue area. Collagen deposition was assessed in various tissue sections, with Bio-Ag NPs(L)@PAN and Bio-Ag NPs(B)@PAN demonstrating the highest levels. The findings from **Table S7** show a significant decrease in SD from days 3 to 14, indicating increasingly uniform collagen distribution over time. This growing consistency highlights the reliability of the collagen deposition process and suggests an adaptive tissue response during maturation. To evaluate skin regeneration at the cellular level and the healing potential of diabetic wounds, histological slices of the defect area were stained with HE on days 3 and 14 post-treatment. Histopathological analysis on day 7 revealed extensive infiltration of inflammatory innate immune cells, such as neutrophils, lymphocytes, and macrophages as well as significant inflammation in the control group wounds. Conversely, the wounds in the Bio-Ag NPs(L)@PAN and Bio-Ag NPs(B)@PAN groups showed enhanced healing, with greater collagen density and a tissue structure resembling normal skin, including well-defined epidermis and stratum spinosum layers. On day 14, all groups reached the proliferation phase, forming an epithelial layer. However, the Bio-Ag NPs(L)@PAN and Bio-Ag NPs(B)@PAN groups displayed an almost fully regenerated and thicker epidermis compared to the control group. The different stages of diabetic wound recovery, as visualized through HE and MT staining, are demonstrated in Fig. 6c and d. MT staining highlighted collagen fibers in blue and subcutaneous muscle fibers in red, revealing a progressive increase in collagen deposition as wounds healed. On day 14, collagen distribution in the control group remained sparse, whereas the Bio-Ag NPs(B)@PAN group exhibited significantly higher collagen deposition with well-developed, tightly packed collagen fibers. Although collagen content in the PAN-treated group was not drastically different, it was considerably higher in the Bio-Ag NPs(L)@PAN and Bio-Ag NPs(B)@PAN groups compared to the control. Collagen, a key component of the extracellular matrix (ECM), plays a crucial role in wound healing by preserving growth factors, enhancing their function, and supplying essential nutrients for tissue regeneration.^56^

### 3.6. Mechanism of diabetic wound healing facilitated by PAN, Bio-Ag NPs(L)@PAN, and Bio-Ag NPs(B)@PAN

**Fig. 7:**
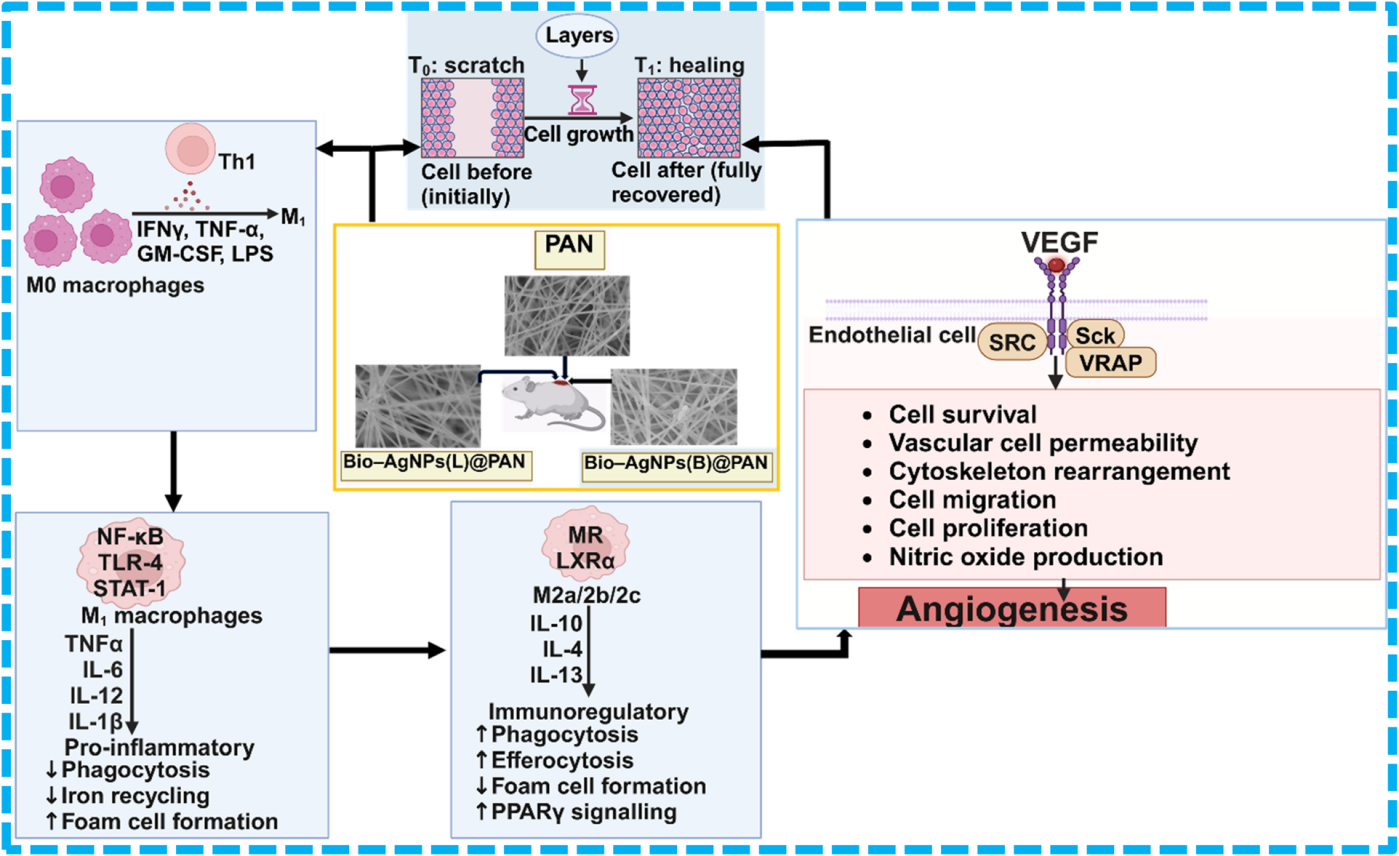
Schematic illustration of the diabetic wound healing mechanism accelerated by Bio-Ag NPs@PAN nanofibers, as well as PAN alone.

The application of Bio-Ag NPs embedded in Polyacrylonitrile (PAN) nanofibers offers a multifunctional approach to accelerate diabetic wound healing. These nanocomposites enhance healing through antibacterial activity, anti-inflammatory effects, enhanced angiogenesis, and improved cell proliferation. Diabetic wounds are highly susceptible to bacterial infections due to high glucose levels that promote microbial growth. Bio-Ag NPs(B)@PAN and Bio-Ag NPs(L)@PAN release silver ions (Ag^+^), which disrupt bacterial cell membranes, inhibit protein synthesis, and induce oxidative stress, effectively eliminating Gram-positive and Gram-negative bacteria. The PAN nanofiber matrix provides a protective barrier against external pathogens while ensuring sustained Ag^+^ release. Chronic inflammation in diabetic wounds delays healing due to excessive pro-inflammatory cytokines (TNF-α, IL-6).^103,104^ Bio-Ag NPs modulate macrophage polarization, shifting them from an inflammatory M_1_ phenotype (pro-inflammatory) to a pro-healing M_2_ phenotype.^105^ This transition reduces oxidative stress and promotes tissue regeneration. Bio-Ag NPs stimulate vascular endothelial growth factor (VEGF) production, which promotes new blood vessel formation (angiogenesis).^106^ Increased angiogenesis improves oxygen and nutrient supply, accelerating granulation tissue formation. PAN nanofibers mimic the extracellular matrix (ECM), create a moist wound environment, and prevent dehydration, providing a scaffold for fibroblast migration and collagen deposition.^107^ The PAN nanofiber network allows for a controlled and sustained release of Bio-Ag NPs, ensuring prolonged antibacterial and regenerative effects. This prevents cytotoxicity due to excessive Ag^+^ release while maintaining a steady therapeutic concentration at the wound site. Therefore, Bio-Ag NPs(L) and Bio-Ag NPs(B) stimulate fibroblast proliferation and upregulate collagen synthesis, promoting faster re-epithelialization and stronger wound closure. Consequently, the application of Bio-Ag NPs(L)@PAN and Bio-Ag NPs(B)@PAN in diabetic wound healing offers a synergistic effect by combating infections through antimicrobial properties, reducing inflammation by modulating macrophage activity, and enhancing angiogenesis to improve oxygen supply.^103,104,108^ The mechanism of diabetic wound healing is illustrated in Fig. 7. The results of this study were compared with previous research on diabetic and non-diabetic wound healing using a variety of materials, as shown in Table 3.

**Table 3:** An overview of previous studies on diabetic and normal wound healing using various materials, provided for comparison with the findings of this study

Post-treatment (days)	Diabetic wound closure figures	References	Remarks
0, 7, 14, 21	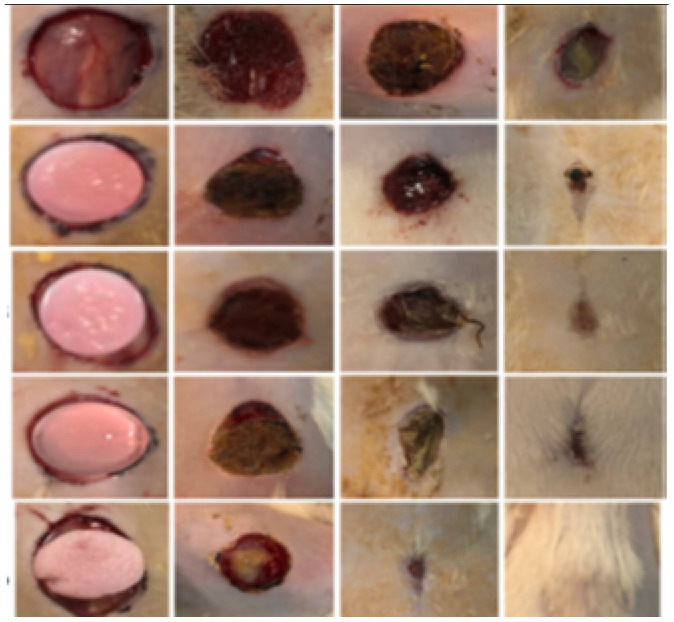	59	Diabetic wound healing
0, 4, 7, 14, 21	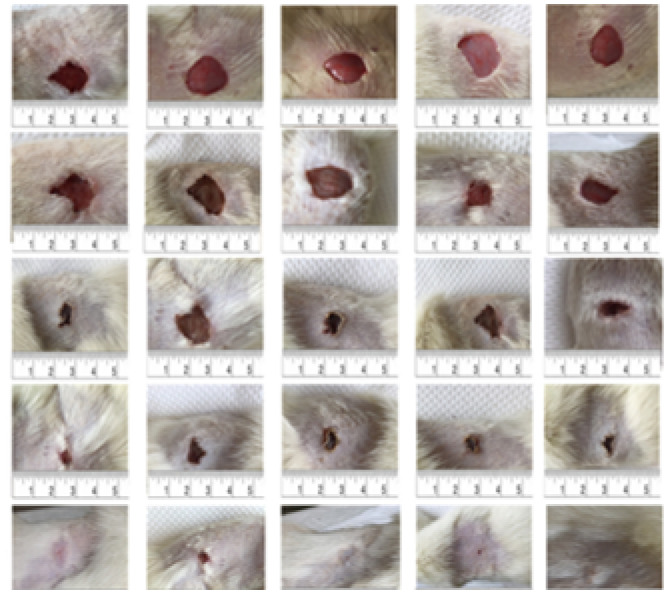	43	Diabetic and normal wound healing
0, 3, 7, 14	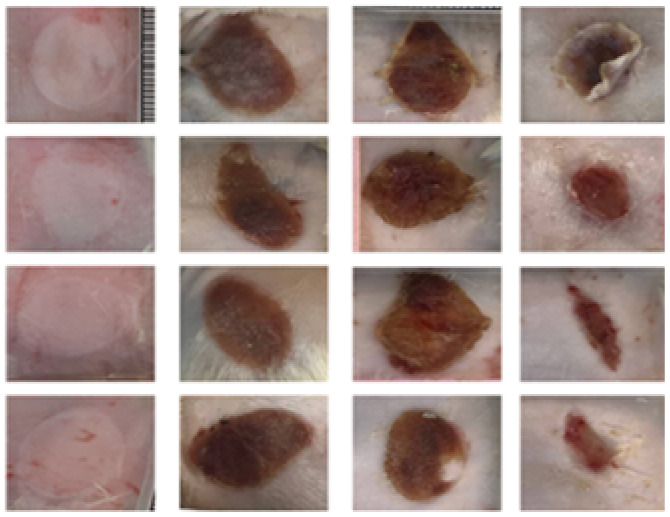	42	Normal wound healing at AGS@AgNPs
0, 3, 7, 11, 14	Fig. 6a	This study	Diabetic wound healing in this study demonstrated significant similarities to findings reported in the literature, highlighting comparable healing patterns and effectiveness
0, 3, 14	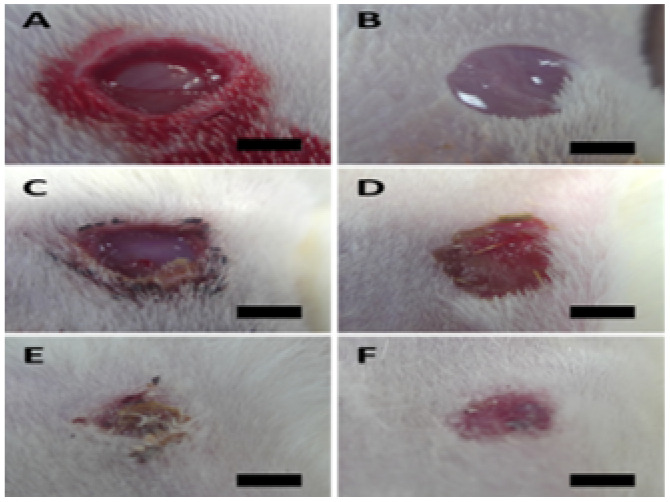	39	Diabetic wound healing
0, 7, 14	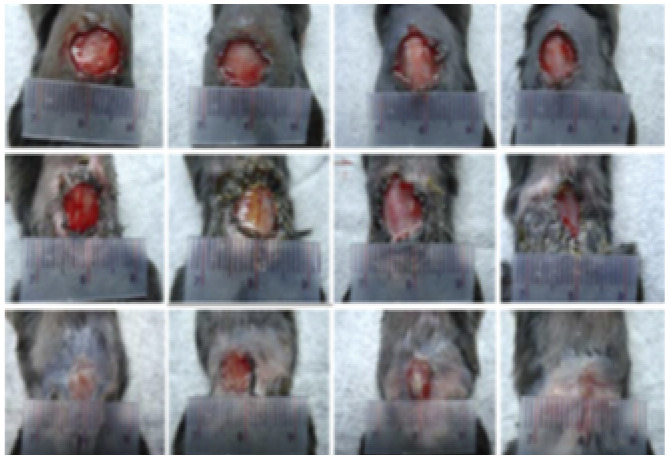	109	Wound healing of diabetic ulcers
−2, 0, 3, 7, 10, 14	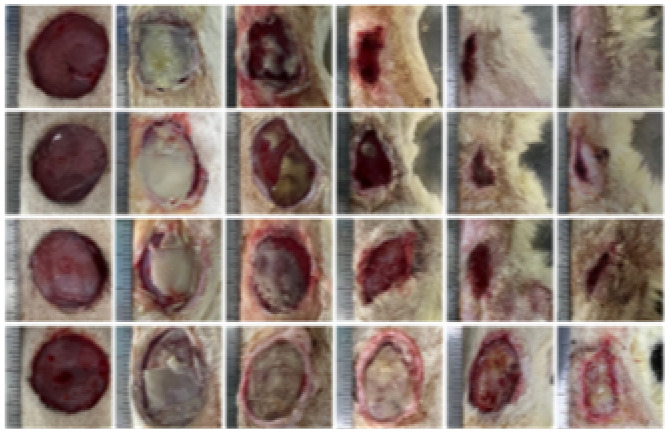	110	Diabetic wound healing
0, 3, 7, 10, 14	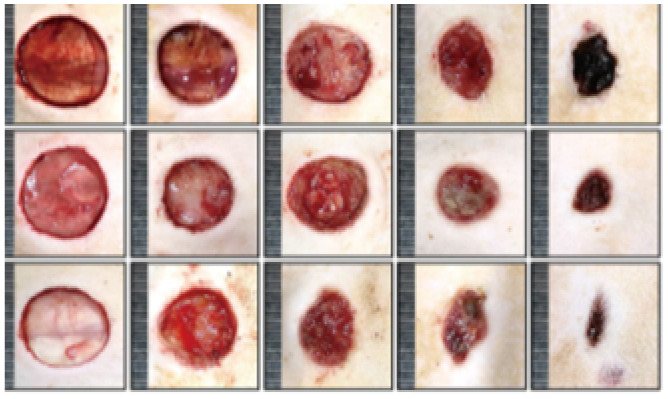	78	Diabetic wound healing

## 4 Conclusion

In this study, Bio-Ag NPs(L) and Bio-Ag NPs(B) were successfully synthesized from the medicinal plant *S. guineense*, along with PAN, Bio-Ag NPs(L)@PAN, and Bio-Ag NPs(B)@PAN nanofibers using the electrospinning method. The successful synthesis and structural integrity were validated through a comprehensive suite of characterization techniques, including UV-vis spectroscopy, FTIR, XRD, SEM, TEM, and EDX analyses. FTIR analysis identified the biomolecules responsible for stabilizing, reducing, and capping the Bio-Ag NPs, and UV-vis spectroscopy verified their synthesis with an absorbance peak at 320 nm. The antibacterial properties of Bio-Ag NPs(L) and Bio-Ag NPs(B) were evaluated at different concentrations, demonstrating significant antibacterial activity. Additionally, both extracts and Bio-Ag NPs exhibited strong intrinsic antioxidant properties, enhancing the bioactivity of plant-derived molecules by scavenging free radicals. At a minimum concentration of 40 mg mL^−1^ and a maximum of 80 mg mL^−1^, the scavenging activity reached 48.53% and 90.60%, respectively. Furthermore, *in vivo* diabetic wound healing studies confirmed the successful synthesis and application of PAN, Bio-Ag NPs(L)@PAN, and Bio-Ag NPs(B)@PAN nanofibers for diabetic wound repair. Bio-Ag NPs(L)@PAN and Bio-Ag NPs(B)@PAN significantly accelerated re-epithelialization and wound closure compared to PAN and the control groups. Histological analysis using HE and Masson's trichrome staining further validated these findings, revealing enhanced collagen deposition in wound tissues. The Bio-Ag NPs(B)@PAN group exhibited the highest collagen density, indicating well-structured and tightly compacted collagen fibers essential for wound healing. This work demonstrated the effective synthesis and application of Bio-Ag NPs and Bio-Ag NPs@PAN electrospun nanofibers in antibacterial, antioxidant, and diabetic wound healing treatments. The research highlights the therapeutic potential of *S. guineense*, a plant rich in natural compounds that can be harnessed to address a wide range of ailments. The findings suggest that this novel approach could pave the way for the development of advanced diabetic wound healing treatments and antimicrobial therapies. Leveraging the antibacterial, anti-inflammatory, and regenerative properties of *S. guineense*-derived Bio-Ag NPs offers a promising solution to the challenges associated with diabetic wound healing. The incorporation of Bio-Ag NPs into PAN nanofibers not only enhances their structural integrity but also improves their functional efficacy, making them suitable for biomedical applications and underscoring the potential of Bio-Ag NPs@PAN nanofibers as a multifunctional therapeutic platform for promoting faster recovery and improving clinical outcomes in multi-aliment management.

## Ethical statement

The study was approved by the Animal Ethics Committee of China-Japan Friendship Hospital (approval number: ZRDWLL240089). All of the procedures used in this animal experiment adhere to the ethical guidelines for the study.

## Author contributions

Teshale Ayano Begeno: writing – review & editing, writing – original draft, visualization, validation, software, methodology, investigation, formal analysis, data curation, conceptualization. Yaqi Zhang: writing – review & editing, formal analysis, data curation. Abdurohman Mengesha Yessuf: writing – review & editing, formal analysis. Tibebu Shiferaw Kassa: writing – review & editing. Ahmed M. Salama: writing – review & editing. Weiguo Wang: writing – review & editing, project administration, funding acquisition. Zhenxia Du: writing – review & editing, supervision, project administration, funding acquisition, formal analysis, data curation, conceptualization.

## Conflicts of interest

Authors hereby declare that we have no financial or personal ties to individuals or groups that could unduly influence this work. Furthermore, authors have no personal or professional stake in any company, product, or service that might be interpreted as influencing the viewpoint expressed in this manuscript. Hence, authors have confirmed no conflict of interests.

## Data Availability

All data provided and/or analyzed during this study were included as figures and tables in this article and its SI. Supplementary information is available. See DOI: https://doi.org/10.1039/d5na00343a.

## References

[cit1] Njenga L., Ayabei K., Akenga T., Onyambu Z., Kiptoo J., Onani M. (2023). Anti-Bacterial Activities of Green Synthesized ZnO and CuO Nanoparticles from Leaf Extracts of *Warburgia ugandensis*. Am. J. Nano Res. Appl..

[cit2] Takele E., Feyisa Bogale R., Shumi G., Kenasa G. (2023). Green Synthesis, Characterization, and Antibacterial Activity of CuO/ZnO Nanocomposite Using Zingiber officinale Rhizome Extract. J. Chem..

[cit3] Tianshuo L., Daxiang C., Tianyuan L., Xinna Y., Meizhen H. (2023). Synthesis, *Nano Biomed. Eng. Appl.*.

[cit4] Vasyliev G., Vorobyova V. (2020). Valorization of Food Waste to Produce Eco-Friendly Means of Corrosion Protection and ‘green’ Synthesis of Nanoparticles. Adv. Mater. Sci. Eng..

[cit5] Shah M. (2014). *et al.*, Gold nanoparticles: Various methods of synthesis and antibacterial applications. Front. Biosci.-Landmark.

[cit6] Hedkvist O. , Synthesis and Characterization of Gold Nanoparticles Degree Project in Engineering Physics, First Level SA104X KTH Division of Functional Materials Written by: Olof Hedkvist, olofhed@kth.se Supervisor: Muhammet Toprak, 2013

[cit7] Verma H. N., Singh P., Chavan R. M. (2014). Gold nanoparticle: Synthesis and characterization. Vet. World.

[cit8] Oliveira A. E. F., Pereira A. C., Resende M. A. C., Ferreira L. F. (2023). Gold Nanoparticles: A Didactic Step-by-Step of the Synthesis Using the Turkevich Method, Mechanisms, and Characterizations. Analytica.

[cit9] Reddy S. J. (2015). Silver nanoparticles - synthesis , applications and toxic effects on humans : a review. Int. J. Bioassays.

[cit10] Kodintcev A. N. (2022). Characterization and potential applications of silver nanoparticles: an insight on different mechanisms. Chim. Techno Acta.

[cit11] Burduniuc O., Bostanaru A. C., Mares M., Biliuta G., Coseri S. (2021). Synthesis, characterization, and antifungal activity of silver nanoparticles embedded in Pullulan matrices. Materials.

[cit12] Pulit-Prociak J., Banach M. (2016). Silver nanoparticles - A material of the future…?. Open Chem..

[cit13] Stepanov A. L., Golubev A. N., Nikitin S. I., Osin Y. N. (2014). A review on the fabrication and properties of platinum nanoparticles. Rev. Adv. Mater. Sci..

[cit14] Cueto M., Sanz M., Oujja M., Gámez F., Martínez-Haya B., Castillejo M. (2011). Platinum nanoparticles prepared by laser ablation in aqueous solutions: Fabrication and application to laser desorption ionization. J. Phys. Chem. C.

[cit15] Fahmy S. A., Preis E., Bakowsky U., Azzazy H. M. E. S. (2020). Platinum Nanoparticles: Green Synthesis and Biomedical Applications. Molecules.

[cit16] Jeyaraj M., Gurunathan S., Qasim M., Kang M. H., Kim J. H. (2019). A comprehensive review on the synthesis, characterization, and biomedical application of platinum nanoparticles. Nanomaterials.

[cit17] Joudeh N., Saragliadis A., Koster G., Mikheenko P., Linke D. (2022). Synthesis methods and applications of palladium nanoparticles: A review. Frontal Nanotechnol..

[cit18] Edayadulla N., Basavegowda N., Lee Y. R. (2015). Green synthesis and characterization of palladium nanoparticles and their catalytic performance for the efficient synthesis of biologically interesting di(indolyl)indolin-2-ones. J. Ind. Eng. Chem..

[cit19] Sahin M., Gubbuk I. H. (2022). Green synthesis of palladium nanoparticles and investigation of their catalytic activity for methylene blue, methyl orange and rhodamine B degradation by sodium borohydride. React. Kinet., Mech. Catal..

[cit20] Bhagat M., Anand R., Sharma P., Rajput P., Sharma N., Singh K. (2021). Review—Multifunctional Copper Nanoparticles: Synthesis and Applications. ECS J. Solid State Sci. Technol..

[cit21] Satyvaldiev A. S. (2018). *et al.*, Copper Nanoparticles: Synthesis and Biological Activity. IOP Conf. Ser.: Mater. Sci. Eng..

[cit22] Amaliyah S., Pangesti D. P., Masruri M., Sabarudin A., Sumitro S. B. (2020). Green synthesis and characterization of copper nanoparticles using Piper retrofractum Vahl extract as bioreductor and capping agent. Heliyon.

[cit23] Sánchez-Sanhueza G., Fuentes-Rodríguez D., Bello-Toledo H. (2016). Copper Nanoparticles as Potential Antimicrobial Agent in Disinfecting Root Canals: A Systematic Review. Int. J. Odontostomatol..

[cit24] Gupta S. (2017). *et al.*, Management of Chronic Wounds: Diagnosis, Preparation, Treatment, and Follow-up. Wounds.

[cit25] Hait M. (2019). Research Trends in Medicinal Plant Sciences. Res. Trends Med. Plant Sci..

[cit26] John S. T., Gungsat N. J., Mamman W. B., Philomena A., Geofrey S. (2019). Synthesis of Silver Nano Particles and Antimicrobial Activities Using Stem Bark Extract of Water Berry (Syzygium Guineense). International Journal of Trend in Research and Development.

[cit27] Orwa C. , Mutua A., Kindt R., Jamnadass R. and Simons A., Agroforestree Database a Tree Reference Selection Guide Version, ORWA et al. pdf, 2009, vol. 4

[cit28] Kiruthiga K., Saranya J., Eganathan P., Sujanapal P., Parida A. (2011). Chemical Composition, Antimicrobial, Antioxidant and Anticancer Activity of Leaves of Syzygium benthamianum (Wight ex Duthie) Gamble. J. Biol. Act. Prod. Nat..

[cit29] Zhou Y. (2023). *et al.*, Urinary Tract Infections Caused by Uropathogenic Escherichia coli: Mechanisms of Infection and Treatment Options. Int. J. Mol. Sci..

[cit30] Mandracchia V. J., Hayes D. W., Yoho R. M., Hayes M. F. (2000). Diagnosis, Differential and Treatment Options. Nat. Rev. Microbiol..

[cit31] Loha M., Mulu A., Abay S. M., Ergete W., Geleta B. (2019). Acute and Subacute Toxicity of Methanol Extract of Syzygium guineense Leaves on the Histology of the Liver and Kidney and Biochemical Compositions of Blood in Rats. Evidence-Based Complementary Altern. Med..

[cit32] Mesfin F., Seta T., Assefa A. (2014). An ethnobotanical study of medicinal plants in Amaro Woreda, Ethiopia. Ethnobot. Res. Appl..

[cit33] Desalegn T., Murthy H. C. A., Ravikumar C. R., Nagaswarupa H. P. (2021). Green Synthesis of CuO Nanostructures using Syzygium guineense (Willd.) DC Plant Leaf Extract and Their Applications. J. Nanostruct..

[cit34] Li K., Zhu Z., Zhai Y., Chen S. (2023). Recent Advances in Electrospun Nanofiber-Based Strategies for Diabetic Wound Healing Application. Pharmaceutics.

[cit35] Yazdanpanah L. (2015). Literature review on the management of diabetic foot ulcer. World J. Diabetes.

[cit36] Abazari M., Ghaffari A., Rashidzadeh H., Badeleh S. M., Maleki Y. (2022). A Systematic Review on Classification, Identification, and Healing Process of Burn Wound Healing. Int. J. Low. Extrem. Wounds.

[cit37] Swearingen B. J., Graves S. S., Storb R., Mathes D. W. (2019). Abstract 47: AMD3100 (Plerixafor) As A Single-Dose Stem Cell Mobilizing Agent In Vascularized Composite Tissue Allograft (VCA) Transplantation In A Canine DLA-Mismatch Model. Plast. Reconstr. Surg. Glob. Open.

[cit38] Wei Y. J. (2024). *et al.*, Kill Two Birds with One Stone: Dual-Metal MOF-Nanozyme-Decorated Hydrogels with ROS-Scavenging, Oxygen-Generating, and Antibacterial Abilities for Accelerating Infected Diabetic Wound Healing. Small.

[cit39] Lee C. H., Huang S. C., Hung K. C., Cho C. J., Liu S. J. (2022). Enhanced Diabetic Wound Healing Using Electrospun Biocompatible PLGA-Based Saxagliptin Fibrous Membranes. Nanomaterials.

[cit40] Armstrong D. G., Swerdlow M. A., Armstrong A. A., Conte M. S., Padula W. V., Bus S. A. (2020). Five year mortality and direct costs of care for people with diabetic foot complications are comparable to cancer. J. Foot Ankle Res..

[cit41] Shaikh-Kader A., Houreld N. N., Rajendran N. K., Abrahamse H. (2019). The link between advanced glycation end products and apoptosis in delayed wound healing. Cell Biochem. Funct..

[cit42] Zare-bidaki M., Mohammadparast-tabas P. (2024). Bio-synthesized AGS @ AgNPs for wound healing, antioxidant support, antibacterial defense, and anticancer intervention. Biocatal. Agric. Biotechnol..

[cit43] Younis N. S., Mohamed M. E., El Semary N. A. (2022). Green Synthesis of Silver Nanoparticles by the Cyanobacteria Synechocystis sp.: Characterization, Antimicrobial and Diabetic Wound-Healing Actions. Mar. Drugs.

[cit44] Sarwar M. N. (2021). *et al.*, Evaluating antibacterial efficacy and biocompatibility of pan nanofibers loaded with diclofenac sodium salt. Polymers.

[cit45] Huang C., Xu X., Fu J., Yu D. G., Liu Y. (2022). Recent Progress in Electrospun Polyacrylonitrile Nanofiber-Based Wound Dressing. Polymers.

[cit46] Chen P. (2022). *et al.*, Electrospinning polyacrylonitrile (PAN) based nanofiberous membranes synergic with plant antibacterial agent and silver nanoparticles (AgNPs) for potential wound dressing. Mater. Today Commun..

[cit47] Serag E., El-Aziz A. M. A., El-Maghraby A., Taha N. A. (2022). Electrospun non-wovens potential wound dressing material based on polyacrylonitrile/chicken feathers keratin nanofiber. Sci. Rep..

[cit48] Nanofibers E. (2018). *et al.*, Antimicrobial and Wound Healing Properties of Polyacrylonitrile-Moringa Antimicrobial and Wound Healing Properties of Polyacrylonitrile- Moringa Extract Nano fi bers. ACS Omega.

[cit49] Huang C., Xu X., Fu J., Yu D. (2022). Recent Progress in Electrospun Polyacrylonitrile Nanofiber-Based Wound Dressing. Polymers.

[cit50] He M. (2020). *et al.*, Electrospun silver nanoparticles-embedded feather keratin/poly(vinyl alcohol)/poly(ethylene oxide) antibacterial composite nanofibers. Polymers.

[cit51] Iravani S. (2011). Green synthesis of metal nanoparticles using plants. Green Chem..

[cit52] Akhtar M. S., Panwar J., Yun Y. (2013). Biogenic Synthesis of Metallic Nanoparticles by Plant Extracts. ACS Sustainable Chem. Eng..

[cit53] Shaheen T. I., Abdelhameed M. F., Zaghloul S., Montaser A. S. (2022). In vivo assessment of the durable, green and in situ bio-functional cotton fabrics based carboxymethyl chitosan nanohybrid for wound healing application. Int. J. Biol. Macromol..

[cit54] Qi J. (2022). *et al.*, Synthesis of silver/Fe3O4@chitosan@polyvinyl alcohol magnetic nanoparticles as an antibacterial
agent for accelerating wound healing. Int. J. Biol. Macromol..

[cit55] Aljohani M. M. (2023). *et al.*, One-pot microwave synthesis of chitosan-stabilized silver nanoparticles entrapped polyethylene oxide nanofibers, with their intrinsic antibacterial and antioxidant potency for wound healing. Int. J. Biol. Macromol..

[cit56] Fu X. (2023). *et al.*, A novel antibacterial hydrogel based on thiolated ovalbumin/gelatin with silver ions to promote wound healing in mice. Int. J. Biol. Macromol..

[cit57] Zhou C. (2024). *et al.*, AgNPs loaded adenine-modified chitosan composite POSS-PEG hybrid hydrogel with enhanced antibacterial and cell proliferation properties for promotion of infected wound healing. Int. J. Biol. Macromol..

[cit58] Dugam S., Jain R., Dandekar P. (2024). Silver nanoparticles loaded triple-layered cellulose-acetate based multifunctional dressing for wound healing. Int. J. Biol. Macromol..

[cit59] Bashiri Z. (2024). *et al.*, In-vitro and in-vivo evaluation of angiogenic potential of a novel lithium chloride loaded silk fibroin/alginate 3D porous scaffold with antibacterial activity, for promoting diabetic wound healing. Int. J. Biol. Macromol..

[cit60] Li R., Chen Z., Ren N., Wang Y., Wang Y., Yu F. (2019). Biosynthesis of silver oxide nanoparticles and their photocatalytic and antimicrobial activity evaluation for wound healing applications in nursing care. J. Photochem. Photobiol., B.

[cit61] Dessalegn E., Mengisteab M., Hiwot G., Nigatu T. (2023). Determination of total phenolic and flavonoid contents, antioxidant and antibacterial potential of the bark extracts of Syzygium guineense (Wild.) DC. Res. Sqaure.

[cit62] Mavanza S. A., Omwenga G. I., Ngugi M. P. (2023). Antibacterial and phytochemical effects of ethanol extracts of Syzygium guineense (Willd.) DC barks and Mangifera indica L seeds. J. Adv. Biotechnol. Exp. Ther..

[cit63] Chirchir D. K., Cheplogoi P. K., Omolo J. O. (2019). Chemical characterization of Syzygium guineense (Myrtaceae) stem bark extracts. J. Pharmacogn. Phytochem..

[cit64] Zhang Q. W., Lin L. G., Ye W. C. (2018). Techniques for extraction and isolation of natural products: A comprehensive review. Chin. Med..

[cit65] Chemat F., Rombaut N., Sicaire A. G., Meullemiestre A., Fabiano-Tixier A. S., Abert-Vian M. (2017). Ultrasound assisted extraction of food and natural products. Mechanisms, techniques, combinations, protocols and applications. A review. Ultrason. Sonochem..

[cit66] Cervantes-Gaxiola M. E., Vázquez-González F. A., Rios-Iribe E. Y., Méndez-Herrera P. F., Leyva C. (2024). Effect of pH on the green synthesis of ZnO nanoparticles using Sorghum bicolor seed extract and their application in photocatalytic dye degradation. Mater. Lett..

[cit67] Alqadi M. K., Abo Noqtah O. A., Alzoubi F. Y., Alzouby J., Aljarrah K. (2014). PH effect on the aggregation of silver nanoparticles synthesized by chemical reduction. Mater. Sci.-Pol..

[cit68] Krishnaraj C., Jagan E. G., Rajasekar S., Selvakumar P., Kalaichelvan P. T., Mohan N. (2010). Synthesis of silver nanoparticles using Acalypha indica leaf extracts and its antibacterial activity against water borne pathogens. Colloids Surf., B.

[cit69] Shukla V. K., Pandey S., Pandey A. C. (2010). Green synthesis of silver nanoparticles using neem leaf (Azadirachta indica) extract. AIP Conf. Proc..

[cit70] Lalitha A., Subbaiya R., Ponmurugan P. (2013). Green synthesis of silver nanoparticles from leaf extract Azhadirachta indica and to study its anti-bacterial and antioxidant property. Int. J. Curr. Microbiol. Appl. Sci..

[cit71] Philip D., Unni C. (2011). Extracellular biosynthesis of gold and silver nanoparticles using Krishna tulsi (Ocimum sanctum) leaf. Phys. E.

[cit72] Sadeghi B., Gholamhoseinpoor F. (2015). A study on the stability and green synthesis of silver nanoparticles using Ziziphora tenuior (Zt) extract at room temperature. Spectrochim. Acta, Part A.

[cit73] Banerjee P., Satapathy M., Mukhopahayay A., Das P. (2014). Leaf extract mediated green synthesis of silver nanoparticles from widely available Indian plants: Synthesis, characterization, antimicrobial property and toxicity analysis. Bioresour. Bioprocess..

[cit74] Desalegn T., Murthy A., Adimasu Y. (2020). Nano sized Fe-Al mixed oxide with natural maize cob sorbent for lead remediation View project Nanomaterials for Adsorption and Antibacterial activity View project ASTU Medicinal Plant Syzygium Guineense (Willd.) DC Leaf Extract Mediated Green Synthesis of Ag Nanoparticles: Investigation of their Antibacterial Activity. Ethiop. J. Sci. Sustain. Dev..

[cit75] Nayak B., Roy S., Roy M., Mitra A., Karak K. (2018). Phytochemical, antioxidant and antimicrobial screening of suaeda maritima L (Dumort) against human pathogens and multiple drug resistant bacteria. Indian J. Pharm. Sci..

[cit76] Yin L. (2023). *et al.*, Sequential Anti-Infection and Proangiogenesis of DMOG@ZIF-8/Gelatin-PCL Electrospinning Dressing for Chronic Wound Healing. ACS Appl. Mater. Interfaces.

[cit77] Wang Y. (2024). *et al.*, Versatile dopamine-functionalized hyaluronic acid-recombinant human collagen hydrogel promoting diabetic wound healing via inflammation control and vascularization tissue regeneration. Bioact. Mater..

[cit78] Hu Y. (2021). *et al.*, Exosomes derived from pioglitazone-pretreated MSCs accelerate diabetic wound healing through enhancing angiogenesis. J. Nanobiotechnol..

[cit79] Das P., Dutta T., Manna S., Loganathan S., Basak P. (2022). Facile green synthesis of non-genotoxic, non-hemolytic organometallic silver nanoparticles using extract of crushed, wasted, and spent Humulus lupulus (hops): Characterization, anti-bacterial, and anti-cancer studies. Environ. Res..

[cit80] Kundu S., Nithiyanantham U. (2013). *In situ* formation of curcumin stabilized shape-selective Ag nanostructures in aqueous solution and their pronounced SERS activity. RSC Adv..

[cit81] Rajput S., Kumar D., Agrawal V. (2020). Green synthesis of silver nanoparticles using Indian Belladonna extract and their potential antioxidant, anti-inflammatory, anticancer and larvicidal activities. Plant Cell Rep..

[cit82] Wan Mat Khalir W. K. A., Shameli K., Jazayeri S. D., Othman N. A., Che Jusoh N. W., Hassan N. M. (2020). Biosynthesized Silver Nanoparticles by Aqueous Stem Extract of Entada spiralis and Screening of Their Biomedical Activity. Front. Chem..

[cit83] Meena P. L., Poswal K., Surela A. K., Saini J. (2021). Facile synthesis of ZnO/CuO/Ag2O ternary metal oxide nanocomposite for effective photodegradation of organic water pollutants. Water Sci. Technol..

[cit84] Zhou W., Liu H., Wang J., Liu D., Du G., Cui J. (2010). Ag2O/TiO2 nanobelts heterostructure with enhanced ultraviolet and visible photocatalytic activity. ACS Appl. Mater. Interfaces.

[cit85] Wang X., Li S., Yu H., Yu J., Liu S. (2011). Ag2O as a new visible-light photocatalyst: Self-stability and high photocatalytic activity. Chem.–Eur. J..

[cit86] Edirisinghe D. , Synthesis of Nanoparticles in Silicate Matrix and Stimuli Responsive Nanocomposite Polymer Films, 2025

[cit87] Azzi M. (2024). *et al.*, Antimutagenic and anticoagulant therapeutic effects of Ag/Ag2O nanoparticles from Olea europaea leaf extract: mitigating metribuzin-induced hepato-and nephrotoxicity. Front. Pharmacol.

[cit88] Laouini S. E. (2021). *et al.*, Green synthesized of ag/ag2o nanoparticles using aqueous leaves extracts of phoenix dactylifera l. And their azo dye photodegradation. Membranes.

[cit89] Durán N., Durán M., de Jesus M. B., Seabra A. B., Fávaro W. J., Nakazato G. (2016). Silver nanoparticles: A new view on mechanistic aspects on antimicrobial activity. Nanomed.: Nanotechnol. Biol. Med..

[cit90] Villarreal-Gómez L. J. (2021). *et al.*, Antimicrobial effect of electrospun nanofibers loaded with silver nanoparticles: Influence of Ag incorporation method. J. Nanomater..

[cit91] Yin I. X., Zhang J., Zhao I. S., Mei M. L., Li Q., Chu C. H. (2020). The antibacterial mechanism of silver nanoparticles and its application in dentistry. Int. J. Nanomed..

[cit92] Bapat R. A. (2018). *et al.*, An overview of application of silver nanoparticles for biomaterials in dentistry. Mater. Sci. Eng., C.

[cit93] Khorrami S., Zarrabi A., Khaleghi M., Danaei M., Mozafari M. R. (2018). Selective cytotoxicity of green synthesized silver nanoparticles against the MCF-7 tumor cell line and their enhanced antioxidant and antimicrobial properties. Int. J. Nanomed..

[cit94] Liao C., Li Y., Tjong S. C. (2019). Bactericidal and cytotoxic properties of silver nanoparticles. Int. J. Mol. Sci..

[cit95] Phull A.-R. (2016). *et al.*, Antioxidant, cytotoxic and antimicrobial activities of green synthesized silver nanoparticles from crude extract of Bergenia ciliata. Future J. Pharm. Sci..

[cit96] Anbu P., Gopinath S. C. B., Yun H. S., Lee C. G. (2019). Temperature-dependent green biosynthesis and characterization of silver nanoparticles using balloon flower plants and their antibacterial potential. J. Mol. Struct..

[cit97] Salmen S. H., Alharbi S. A. (2020). Silver nanoparticles synthesized biogenically from Aloe fleurentiniorum extract: characterization and antibacterial activity. Green Chem. Lett. Rev..

[cit98] Abdel-Aziz M. S., Shaheen M. S., El-Nekeety A. A., Abdel-Wahhab M. A. (2014). Antioxidant and antibacterial activity of silver nanoparticles biosynthesized using Chenopodium murale leaf extract. J. Saudi Chem. Soc..

[cit99] Jain S., Mehata M. S. (2017). Medicinal Plant Leaf Extract and Pure Flavonoid Mediated Green Synthesis of Silver Nanoparticles and their Enhanced Antibacterial Property. Sci. Rep..

[cit100] Ibrahim H. M. M. (2015). Green synthesis and characterization of silver nanoparticles using banana peel extract and their antimicrobial activity against representative microorganisms. J. Radiat. Res. Appl. Sci..

[cit101] Kalaimurgan D. (2023). *et al.*, Biogenic synthesis of zinc oxide nanoparticles using Drynaria Quercifolia tuber extract for antioxidant, antibiofilm, larvicidal, and photocatalytic applications. Biomass Convers. Biorefin..

[cit102] Uza N. U. (2024). *et al.*, Green Synthesis, Characterization and Pharmaceutical Applications of Biocompatible Zinc Oxide Nanoparticles Using Heliotropium rariflorum Stocks. Pharmaceuticals.

[cit103] Rodrigues M., Kosaric N., Bonham C. A., Gurtner G. C. (2019). Wound healing: A cellular perspective. Physiol. Rev..

[cit104] Velnar T., Bailey T., Smrkolj V. (2009). The wound healing process: An overview of the cellular and molecular mechanisms. J. Int. Med. Res..

[cit105] Krzyszczyk P., Schloss R., Palmer A., Berthiaume F. (2018). The role of macrophages in acute and chronic wound healing and interventions to promote pro-wound healing phenotypes. Front. Physiol..

[cit106] Hart J. (1998). Inflammation. 2: Its role in the healing of chronic wounds. J. Wound Care.

[cit107] Kular J. K., Basu S., Sharma R. I. (2014). The extracellular matrix: Structure, composition, age-related differences, tools for analysis and applications for tissue engineering. J. Tissue Eng..

[cit108] Pastar I. (2014). *et al.*, Epithelialization in Wound Healing: A Comprehensive Review. Adv. Wound Care.

[cit109] Choi J. S., Leong K. W., Yoo H. S. (2008). In vivo wound healing of diabetic ulcers using electrospun nanofibers immobilized with human epidermal growth factor (EGF). Biomaterials.

[cit110] Hu D. (2024). *et al.*, Accelerated healing of intractable biofilm-infected diabetic wounds by trypsin-loaded quaternized chitosan hydrogels that disrupt extracellular polymeric substances and eradicate bacteria. Int. J. Biol. Macromol..

